# Physiological relevance of autocrine melatonin signaling in pineal and extrapineal sites: a systematic review

**DOI:** 10.1152/function.101.2025

**Published:** 2026-01-19

**Authors:** Gaudence Ndinganire, Gaspard Ntamukunzi, Abdullateef Isiaka Alagbonsi

**Affiliations:** Department of Physiology, School of Medicine and Pharmacy, College of Medicine and Health Sciences, University of Rwanda, Huye, Rwanda

**Keywords:** autocrine, extrapineal melatonin, melatonin, paracrine, pineal gland

## Abstract

Beyond the pineal gland, melatonin is produced locally in many extrapineal organs, where it mediates local tissue homeostasis. However, little attention is paid to the physiological role of autocrine melatonin signaling across body organs. This study synthesizes original data to address this gap, as gaining insight into this topic could lead to new therapeutic approaches for diseases associated with melatonin. This systematic review used a narrative synthesis, following the Preferred Reporting Items for Systematic Reviews and Meta-Analyses (PRISMA) 2020 guideline standards to synthesize original articles published between January 2000 and July 2025, using the Wiley Online Library and PubMed databases. From the 41 studies reviewed, various targets for the physiological relevance of autocrine melatonin signaling in pineal and extrapineal sites were noted. In descending order, the targets were immunoregulatory switch (8 studies), ovary and reproductive system (7 studies), pineal gland (6 studies), gut (5 studies), skin and hair follicles (3 studies), retina (3 studies), testes (3 studies), liver and metabolic tissues (2 studies), bone (2 studies), cardiovascular/endothelial compartment (1 study), and mitochondria (1 study). A layer of melatoninergic biology that is different from the traditional pineal endocrine signal and has biological and clinical significance is autocrine melatonin signaling in the pineal and numerous extrapineal locations. Although there is a translational potential, thorough mechanistic human research and better assays are required due to model heterogeneity and scarcity of human data.

## Graphical abstract

**Figure:**
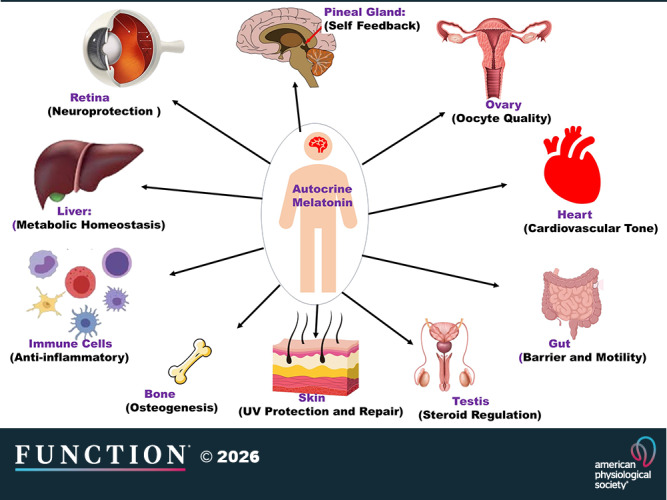

## INTRODUCTION

Melatonin, estimated to be 2.5 billion years old (1), is widely produced across organism species, ranging from prokaryotes (archaea, cyanobacteria, and bacteria), unicellular eukaryotes, and multicellular organisms like plants, fungi, and all animals (2, 3). Diurnal cycle, seasonal biology, and circadian rhythms are all substantially controlled by melatonin, a highly conserved indoleamine that is mostly synthesized and secreted by the pineal gland (4). Historically, melatonin was viewed strictly as an endocrine hormone produced by the pineal gland and released into the bloodstream to regulate the sleep-wake cycle. However, recent research has revealed that melatonin is synthesized in many extrapineal tissues, like the skin (5–7), retina, ovaries, bone marrow, gastrointestinal tract (GIT), immune cells, and placenta (8). Melatonin seems to participate in the local regulation of metabolism, oxidative defense, immunological responses, and cell function (9). Beyond animals, the presence of melatonin and its involvement in many physiological and biochemical processes in plants has been widely studied, though the absence of melatonin in plants from some geographic locations is also established (10).

Cellular apoptosis, proliferation, mitochondrial homeostasis, and redox balance involve autocrine melatonin regulation, which is aided by molecular processes that also facilitate local cellular adjustment of the microenvironment to metabolic and circadian signals (11). Both pineal and extrapineal tissues use G protein-coupled melatonin receptors (MT1 and MT2) as the main mediators of melatonin autocrine signaling (12). Upon binding to these receptors, melatonin induces downstream signaling pathways, including phospholipase C stimulation, which consequently splits phosphatidylinositol biphosphate (PIP_2_) to cause an increase in inositol triphosphate (IP_3_) and diacyl glycerol (DAG). By controlling its own synthesis and receptor expression, melatonin autocrine feedback in the pineal gland modulates the intensity and coordination of the circadian cycle (13). For instance, enzymes like arylalkylamine *N*-acetyltransferase (AANAT) and serotonin *N*-acetyltransferase (SNAT), which are involved in melatonin synthesis, are affected by autocrine melatonin signaling via the MT1 and MT2 on the pinealocytes (14). Beyond MT1 and MT2, melatonin and its metabolites have also been shown to act as agonists of the aryl hydrocarbon receptor (AhR) due to their structural similarity with its natural ligands (15).

A dichotomy in the signal for melatonin secretion in both pineal and extrapineal tissues has been reported. Although the light-dark cycle regulates pineal melatonin secretion, extrapineal tissues have been shown to show little or no dependence on this variation, pointing to the fact that melatonin secretion in these tissues is for local regulation of their physiological functions. For example, mucus and enterochromaffin cells in the gut produce melatonin without the need for light-dark fluctuation, and this secretion affects microbial homeostasis, mucosal immunology, and gastrointestinal motility (16). Melatonin also works on the granulosa cells in the ovary by autocrine MT1/MT2-mediated signaling to intensify follicular maturation, protect against oxidative stress, and control steroidogenesis. Furthermore, melatonin exerts autocrine control over innate and adaptive immune responses, which regulate cytokine production and inflammation (17).

Though its advantages and disadvantages differ depending on the tissue type, exogenous melatonin has a variety of organ-specific effects. Although high dosages may inhibit endogenous melatonin production, melatonin supplements aid in the restoration of circadian regularity, enhance sleep, and lessen neuroinflammation in the brain and pineal gland. Topical melatonin has clinical utility in dermatological diseases by protecting the skin from ultraviolet (UV) damage and hastening wound healing. Although improper timing of delivery may affect gut motility, melatonin improves GIT mucosal healing and decreases its inflammation in inflammatory bowel disease (IBD) (4). Although excessive amounts of melatonin can compromise adaptive immunity, it functions as an immunomodulator inside the immune system by increasing innate immunity and reducing cytokine storms. In the placenta, melatonin reduces oxidative stress and promotes fetal development in complicated pregnancies, though its application in obstetrics remains largely experimental. Notwithstanding these therapeutic values, exogenous melatonin cannot fully replace autocrine melatonin signaling, particularly in tissues where local synthesis targets mitochondria and is not solely mediated by membrane receptors (18). The ameliorative effect of melatonin supplements in various disease conditions associated with oxidative stress is well-documented (19–28).

Notably, changes in autocrine melatonin signaling, whether due to receptor polymorphism, dysregulated synthesis, or defects in downstream pathways, have been linked to a variety of diseases such as metabolic syndrome, IBD, neurodegenerative diseases, infertility, and some types of cancer (29). These findings underscore the importance of developing therapies that target melatonin signaling specifically within individual tissues, rather than relying solely on systemic therapies. It may be more effective to focus on treatment that directly influences melatonin activity within specific tissues, thereby reducing patients’ exposure to systemic side effects seen with many available therapies.

Although the attention of most literature is focused on the physiological effects and therapeutic potentials of exogenous melatonin administration, little attention has been paid to the physiological relevance of locally produced melatonin and its autocrine signaling. This systematic review aims to shed light on how locally produced melatonin functions within the pineal and extrapineal tissues to support various physiological functions. Gaining deeper insight into these localized melatonin actions could enhance our understanding of the physiological importance of autocrine melatonin signaling.

## METHODOLOGY

This systematic review used a narrative synthesis, following the Preferred Reporting Items for Systematic Reviews and Meta-Analyses (PRISMA) 2020 guideline standards (30) (Supplemental File S1). The review carefully combined the findings of both clinical research and laboratory experiments to comprehensively explore melatonin’s action at both cellular and systemic levels, particularly in relation to autocrine signaling in the pineal and extrapineal sites.

### Literature Search Strategy

Scientific databases, including Wiley Online Library and PubMed, were extensively searched for peer-reviewed literature. A combination of the following keywords was used as search terms: “autocrine melatonin,” “melatonin signaling,” “pineal gland,” “extrapineal melatonin,” “melatonin MT1/MT2 Receptors,” and “melatonin and disease.” Melatonin in the specific body tissues was also separately searched.

### Eligibility Criteria

Only articles that met the following criteria were included in this systematic review *1*) peer-reviewed original journal articles, *2*) articles that examine the physiological and/or pathological functions of autocrine or paracrine melatonin signaling in pineal or extrapineal tissues, *3*) studies conducted in mammalian systems, involving either human subjects or animal models (such as mice, rats, and primates), and *4*) articles written in English and published between January 2000 and July 2025. Studies that fall within the following descriptions were excluded: *1*) research that investigated only the release and importance of melatonin from the pineal or extrapineal tissues without any link to the autocrine melatonin signaling and *2*) nonoriginal studies like review articles (e.g., systematic reviews and meta-analysis), conference abstracts, books, opinion articles, editorials, etc.

After abstracts and titles screening, the whole texts of the retrieved studies were evaluated by two reviewers, and any disagreement was settled either by consensus or by arbitration of a third reviewer. Risk of bias assessment was done for the studies using the SYstematic Review Center for Laboratory animal Experimentation (SYRCLE)’s RoB tool for animals (31) and Cochrane RoB 2 for humans (32) (Supplemental File S2).

### Data Extraction and Synthesis

For each study included in this review, the following pieces of information were extracted using a structured data extraction form: Author name, publication year, title of the article, experimental model, medium studied, sample size, type of melatonin manipulation employed, method used in the assay, key findings reported, mechanisms involved in the outcomes, and DOI of the studies (Supplemental File S3). Based on the identified mechanisms and findings, the studies were grouped into the following categories: *1*) mitochondria; *2*) cardiovascular/endothelial compartment; *3*) bone; *4*) liver and metabolic tissues; *5*) testes; *6*) retina; *7*) skin, hair follicles, and peripheral tissues; *8*) gut; *9*) pineal gland; *10*) immunoregulatory switch; *11*) ovary and reproductive system; and *12*) multisystem and experimental support.

## RESULTS

### Article Selection

**Figure 1.**
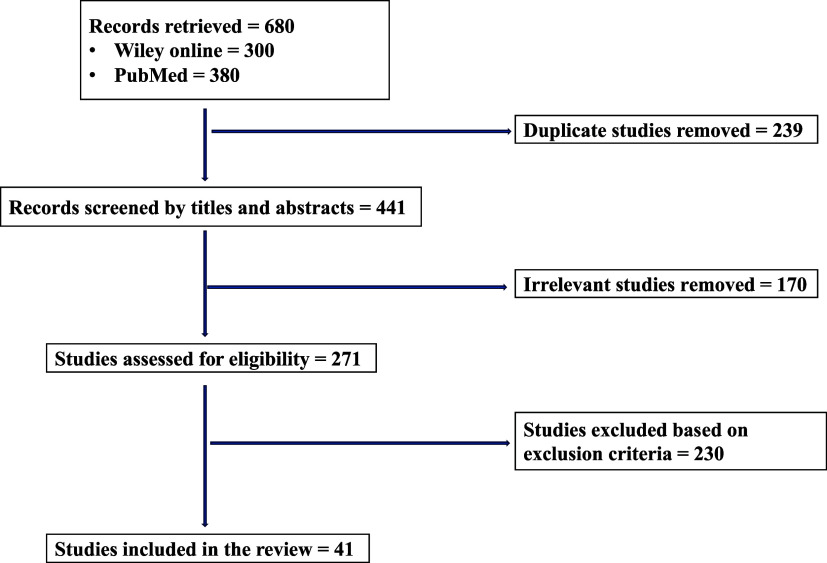
PRISMA flow chart of the article screening and selection process. PRISMA, Preferred Reporting Items for Systematic Reviews and Meta-Analyses.

After the extensive search of the databases, a total of 680 articles were identified, including 300 from Wiley Online and 380 from PubMed. Then, 239 duplicate articles and 170 irrelevant articles were removed, thereby remaining with 271 articles that were screened for eligibility. Out of the screened articles, only 41 fully met the inclusion criteria for this review (Fig. 1).

### Autocrine Melatonin Signaling in Various Organ Systems

**Figure 2.**
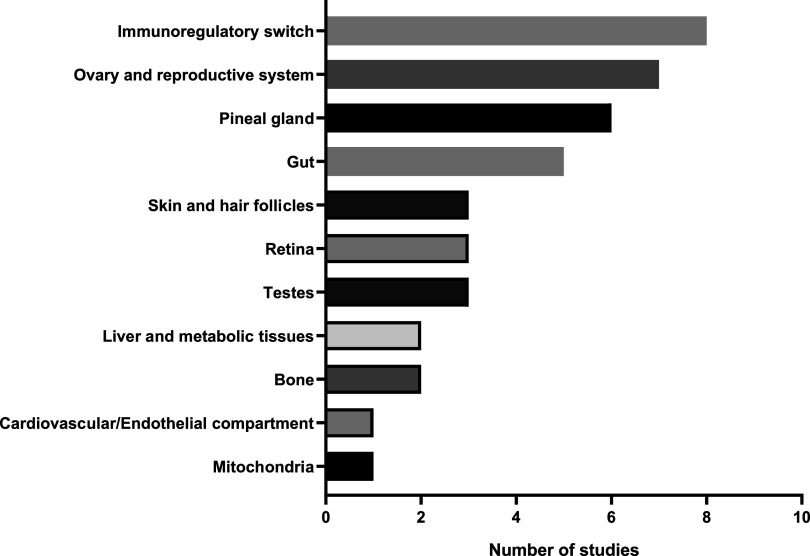
Mechanistic classification of included studies that reported autocrine/paracrine melatonin signaling in various tissues.

From the 41 studies reviewed, various targets for the physiological relevance of autocrine melatonin signaling in pineal and extrapineal sites were noted. In descending order, the targets were immunoregulatory switch (8 studies), ovary and reproductive system (7 studies), pineal gland (6 studies), gut (5 studies), skin and hair follicles (3 studies), retina (3 studies), testes (3 studies), liver and metabolic tissues (2 studies), bone (2 studies), cardiovascular/endothelial compartment (1 study) and mitochondria (1 study) (Fig. 2). A summary of the tissue-specific roles of autocrine melatonin signaling in mammalian species investigated in the reviewed articles is presented in Table 1.

**Table 1.** Tissue-specific role of autocrine melatonin signaling in mammals

Tissues	Authors	Experimental Model	Key Findings	Mechanisms Involved
Ovary and reproductive system	Zheng et al. (33)	Human granulosa cells from patients with OHSS and control granulosa cells	Melatonin helped to alleviate ovarian hyperstimulation syndrome	Melatonin mitigates oxidative stress-induced apoptosis in granulosa cells of patients with through the SESN2 → AMPK → mTOR → 4EBP1/S6K1 signaling pathway, restoring antioxidant defenses and cellular survival mechanisms
	Yu et al. (34)	Human (patients with PCOS)	In hypoestrogenic individuals with PCOS, melatonin increased oocyte quality and decreased androgen production	Not specified (hormonal modulation study)
	Liu et al. (35)	Granulosa cells (animal/human-derived)	Melatonin modulates autophagy and promotes cell survival and follicular development in granulosa cells	ATG2B- and BMPR1B-dependent mechanisms
	Liu et al. (36)	Rat (granulosa cells, ovarian damage model)	Melatonin enhanced follicle numbers, improved ovarian weight and index, modulated hormone levels, and prevented granulosa cell autophagy and ovarian damage	PI3K, Akt, mTOR
	Tamura et al. (37)	Mouse (imprinting control region, ICR strain)	Long-term melatonin therapy enhanced ovarian rejuvenation markers, increased follicle counts, improved fertilization and blastocyst rates, and increased ovulated oocytes	SIRT1, SIRT3, LC3
	Zhang et al. (38)	Female mice (WT and MT1 knockout)	Melatonin supplementation (optimal at 10^−5^ mol/L) significantly increased litter size in 24-wk-old mice. Melatonin increased total oocyte number and improved their quality (more morphologically normal oocytes, higher blastocyst formation rate after IVF) compared with controls. Oocytes from melatonin-treated mice had lower ROS levels and better mitochondrial membrane potential	Through the MT1 → activation of the AMPK pathway, with downstream effects including increased antioxidant capacity, reduced ROS, maintained mitochondrial membrane potential, increased SIRT1, cellular *MYC*, and CHOP expression changes
	Xu et al. (39)	Bovine granulosa cells (in vitro)	Melatonin promoted mitophagy and reduced H_2_O_2_-induced apoptosis in granulosa cells	Melatonin → SIRT1/FoxO1 → ↑mitophagy, ↓apoptosis
Immunoregulatory switch	Muxel et al. (40)	Murine macrophage cell line RAW 264.7 (in vitr*o*)	Macrophage-derived melatonin acts autocrinally to potentiate macrophage phagocytic activity; conversely, luzindole (melatonin receptor antagonist) reduces phagocytosis. The study highlights a switch during inflammation in the source of melatonin from pineal (endocrine) to immune cells (paracrine/autocrine)	LPS and other proinflammatory stimuli activate NF-κB in RAW 264.7 cells and induce transcription of the AANAT promoter via κB elements. AANAT expression and melatonin synthesis increase in stimulated macrophages; this induction is blocked by NF-κB inhibitors or siRNA against RelA/c-Rel
	Xu et al. (41)	Nonviral oxidative or inflammatory injury models, including LPS-induced inflammation, high-glucose or oxidative stress challenges, and chemically induced tissue injury, alongside in vitro experiments using primary cells or cell lines exposed to oxidative or inflammatory stress	Melatonin increased antioxidant defense capacity by activating NRF2 pathways, promoting nuclear translocation of NRF2, and upregulating antioxidant enzymes such as GCL, whereas simultaneously suppressing NF-κB-mediated inflammation, reducing levels of IL-1β, IL-6, and TNF-α, thereby decreasing oxidative stress, inflammation, and cellular injury in the target tissues	Melatonin activates the PI3K/Akt → NRF2 signaling pathway, promoting nuclear translocation of NRF2 and increasing transcription of antioxidant genes such as GCL, HO-1, and NQO1, whereas simultaneously inhibiting the NF-κB pathway, leading to decreased transcription of proinflammatory cytokines; together, this combination of enhanced antioxidant defense and suppressed inflammation results in effective tissue protection
	Sun et al. (42)	Rats in the osteoarthritis model and RAW 264.7 macrophages	Melatonin enhances the ability of M2 macrophages to secrete IL-10	Inhibition of the Erk5 signaling pathway
	Hu et al. (43)	In vitro: rat BMSCs	Melatonin reversed TNF-α-induced inhibition of osteogenesis in BMSCs, suppressing inflammation, supporting therapeutic potential for osteoporosis/inflammation-associated bone loss	Melatonin inhibits NF-κB signaling (including TNF-α-induced) in BMSCs → promotes osteogenic gene expression (ALP, Col 1, OCN, OPN, *RUNX2*) and inhibits inflammatory response
	Carrillo-Vico et al. (44)	Primary human lymphocytes (resting and PHA-stimulated) obtained from human donors; in vitro cell culture experiments	First demonstration that human lymphocytes synthesize and release melatonin in vitro; inhibition of melatonin synthesis reduced IL-2 production (restored by exogenous melatonin), supporting a local autocrine/intracrine immunomodulatory role	Human lymphocytes express the enzymatic machinery for melatonin synthesis: TPH, AANAT, and HIOMT. Endogenous lymphocyte-derived melatonin modulates IL-2 production and IL-2 receptor expression—consistent with intracrine/autocrine/paracrine immunoregulatory roles
	Jiménez-Delgado et al. (45)	In vivo: 26 patients diagnosed with Parkinson’s disease (Hoehn and Yahr, *stages 1*–*3*)	Melatonin affects oxidative stress markers, mitochondrial complex I activity, and respiratory control ratio in platelets of patients with Parkinson’s disease	Melatonin administration significantly reduced plasma levels of lipoperoxides, nitric oxide metabolites, and carbonyl groups compared with placebo. Catalase activity was significantly increased in the melatonin group compared with the placebo. Mitochondrial complex I activity and respiratory control ratio were significantly higher in the melatonin group compared with the placebo. No significant changes were observed in membrane fluidity between the melatonin and placebo groups. These findings suggest that melatonin may play a role in mitigating oxidative stress and enhancing mitochondrial function in patients with Parkinson’s disease
	Lardone et al.(46)	Jurkat human T-cell line	Jurkat cells synthesize melatonin; blocking receptors reduced IL-2, and exogenous melatonin rescued the effect.	Melatonin synthesis by T-cells and acts via melatonin membrane/nuclear receptors → IL-2 production
	Pires-Lapa et al. (47)	Primary BM-DCs from BALB/c mice and RAW 264.7 murine macrophage cell line	Catecholamine-mediated β-adrenoceptor activation in macrophages/dendritic cells triggers AANAT expression/phosphorylation and melatonin synthesis. Blocking β-adrenoceptor (propranolol), PKA (H-89), or NF-κB (PDTC, ALLN) prevents the melatonin increase. The phenomenon suggests a pathway by which sympathetic signaling via β-adrenoceptors can induce extrapineal melatonin synthesis in phagocytes	β-adrenergic (β-adrenoceptor) stimulation via catecholamines (adrenaline, noradrenaline, isoprenaline) → increase in intracellular cAMP → activation of PKA → increased expression and phosphorylation of AANAT → melatonin synthesis. Also involves NF-κB nuclear translocation as necessary for AANAT induction in these immune cells
Pineal gland	González et al. (48)	Transfected cells (biochemical/biophysical receptor work) and ex vivo pineal tissue strips (rodent/ovine)	Demonstrated D_4_ receptor heteromerization with adrenergic receptors that can inhibit adrenergic stimulation of melatonin synthesis in a circadian-dependent manner (novel feedback/modulation mechanism)	Receptor-receptor interactions (α1B-D_4_ and β_1_-D_4_ heteromers) provide circadian-dependent modulation of adrenergic signaling and hence melatonin synthesis—a form of local regulation
	Lépinay et al. (13)	Primary ovine (sheep) pinealocytes in culture (ex vivo)	Melatonin receptors present on pinealocytes that negatively regulate melatonin secretion—evidence for an intrapineal autoinhibitory feedback loop via MT receptors	Identified functional MT receptor expression in pinealocytes and that MT receptor blockade (luzindole) increases cAMP and melatonin secretion—supporting negative autoregulation by melatonin receptors
	Chen et al. (49)	Heterologous cell expression systems (receptor signaling) and signaling assays relevant to pineal receptor function	Dissects intracellular signaling downstream of MT_1_/MT_2_—mechanistic basis for how locally produced melatonin could signal via MT receptors (including possible intrapineal signaling routes)	Showed distinct G-protein dependencies for MT_1_ vs MT_2_ ERK activation—provides molecular basis for diverse local melatonin receptor signaling in tissues (including pinealocytes)
	Zatz et al. (50)	Chick pineal glands (cultured and in vivo)	cAMP signaling increases AANAT protein abundance and activity, and light/cAMP changes control the amplitude of melatonin production in the chick pineal	cAMP and light modulate AANAT protein abundance (post-translational control); cAMP stabilizes AANAT protein at night, promoting melatonin synthesis (local pineal regulatory mechanism controlling melatonin rhythm)
	Obsil et al. (51)	Purified AANAT protein complexed with 14-3-3 (structural biochemistry)	Solved the crystal structure of AANAT:14-3-3ζ complex and showed how 14-3-3 binding stabilizes/activates AANAT—a structural mechanism explaining night-time stabilization of the enzyme	14-3-3 proteins bind phosphorylated AANAT and stabilize/activate it—molecular basis for cAMP-dependent posttranslational stabilization of AANAT in pinealocytes (important for nocturnal melatonin synthesis and showing mechanism for intrapineal regulation at the protein level)
	Baltatu et al. (52)	Rat pineal glands (in vivo and in vitro cultured glands); transgenic rats with brain angiotensinogen suppression	Blocking AT1 receptors or reducing brain angiotensinogen reduced pineal serotonin and melatonin precursors and lowered pineal melatonin—evidence for a local angiotensinergic modulation of pineal indole synthesis	Pineal local renin-angiotensin system (AT1 receptors) tonically modulates indole (serotonin → melatonin) synthesis by regulating TPH activity; local angiotensin influences precursor availability and hence pineal melatonin output (local paracrine/autocrine modulation within pineal)
Gut	Sommansson et al. (53)	Rat, ∼30-mm proximal duodenum perfused in situ	Luminal melatonin rapidly lowered duodenal permeability and increased bicarbonate secretion; the receptor antagonist abolished the effect	Melatonin reduces paracellular permeability and increases bicarbonate secretion via melatonin receptors on the duodenal mucosa
	Lin et al. (54)	ICR mice (8-wk-old male), restraint-stress model	Melatonin restores antioxidant capacity and tight junction protein expression and reduces autophagy/oxidative injury—local protective/paracrine effects in gut mucosa	Restraint stress decreased plasma melatonin and impaired mucosal architecture (↓villus height, ↓goblet cells, ↓tight junction proteins) and antioxidant defenses; melatonin pre-treatment reversed these changes, improving barrier integrity and antioxidant measures (supporting gut-intrinsic protective role
	Sun et al. (55)	Male Sprague-Dawley rats with SAP induced by retrograde taurocholate injection.	Melatonin prevented SAP-related mucosal damage, preserved microvilli, and significantly reduced bacterial translocation into blood—showing protective local gut effects	Melatonin protects intestinal mucosa structural integrity (antioxidant, anti-apoptotic), preventing bacterial translocation (paracrine/local mucosal protection)
	Rungratanawanich et al. (56)	Mouse (in vivo) + human intestinal T84 cells and AML12 mouse hepatocytes (in vitro)	Melatonin pretreatment reduced intestinal permeability, preserved junctional protein expression via Sirt1-dependent deacetylation, lowered endotoxemia, and attenuated liver fibrosis; in vitro data support direct epithelial and hepatic protective mechanisms	Melatonin preserves tight junctions and prevents hyper-acetylation of junctional proteins via Sirt1-related deacetylation—gut-intrinsic effect that prevents gut leakiness and downstream liver fibrosis via the gut-liver axis (paracrine/autocrine + systemic)
	Fernández-Gil et al. (57)	Adult male Wistar rats; tongue irradiation model producing remote small-intestine injury	Local melatonin application increased intestinal melatonin content, prevented mitochondrial dysfunction, suppressed inflammasome/NF-κB activation, reduced apoptosis, and preserved intestinal architecture after irradiation	Melatonin reaches gut tissue and protects epithelial mitochondria, reduces oxidative stress and NLRP3/NF-κB signaling, preserves tight junction proteins—local tissue protective/paracrine effects
Skin, hair follicles, and peripheral tissues	Shen Zhihao et al. (58)	Rat skin-flap model	Melatonin improved skin-flap survival: lower tissue water content, higher vascular density, increased antioxidant proteins; effect reversed by NRF2 inhibitor (ML385), confirming melatonin/Nrf2 pathway	Nrf2 activation; angiogenesis; antioxidant enzyme upregulation; reduced inflammation
	Sevilla et al. (59)	Human melanocytes	Melatonin acts in melanocytes to reduce UV-induced oxidative stress and DNA damage. This article reviews mechanistic studies	Melatonin and derivatives reduce UV-induced damage, modulate melanocyte signaling (including MITF)
	Bocheva et al. (60)	Human keratinocytes and melanocytes (and skin models)	Melatonin stimulated Nrf2 expression/ nuclear translocation; enhanced expression of antioxidant enzymes; reduced UVB-induced ROS and oxidative DNA damage (8-OHdG) in keratinocytes/ melanocytes	Activation of Nrf2, induction of antioxidant enzymes (γ-GCS, HO-1, NQO1) in skin cells under UVB stress
Retina	Baba et al. (61)	WT mice and MT1 receptor knockout (*MT1*^−/−^) mice (in vivo)	WT mice showed a clear diurnal rhythm of ERG responses: higher a- and b-wave amplitudes at night compared to day. MT1-knockout mice did not show this night/day difference in ERG amplitudes. Injection of exogenous melatonin during the day reduced scotopic threshold and increased a-/b-wave amplitude in WT mice but had no effect in *MT1*^−/−^ mice. At 12 mo of age, *MT1*^−/−^ mice had significantly fewer photoreceptor nuclei in the outer nuclear layer compared with WT controls; at 18 mo, the loss was more pronounced, and ganglion cell number was also significantly lower in *MT1*^−/−^ mice than in WT. These data support the conclusion that endogenous melatonin signaling via MT1 receptor is physiologically relevant to maintaining retinal function (via ERG responses) and cell viability (photoreceptors and ganglion cells) with ageing	Endogenous night-phase melatonin signaling via the MT1 receptor supports higher scotopic and photopic ERG a- and b-wave amplitudes at night; loss of MT1 abolishes this diurnal difference, and with ageing (12 mo, 18 mo) leads to greater photoreceptor layer thinning (outer nuclear layer) and ganglion cell loss in *MT1*^−/−^ mice
	Atacak et al. (62)	Aged female rats (≈16 mo old) with diabetes induced by intraperitoneal injection of Streptozotocin (STZ). Animals divided into 4 groups: control, control + melatonin, diabetes, diabetes + melatonin	The diabetes group had the highest retinal MDA (marker of lipid peroxidation/oxidative stress), The diabetes + melatonin group had the highest retinal GSH (antioxidant) values and the highest SIRT1 expression values among the groups. The authors concluded that melatonin supplementation exerts a protective effect on retinal tissue in diabetic aged female rats via increasing antioxidant activity and SIRT1 gene expression	Melatonin supplementation → increased retinal antioxidant activity (↑ GSH; ↓ MDA) + induction of retinal SIRT1 gene expression → reduced retinal oxidative damage and structural degeneration in diabetic aged rats
	Jiang et al. (63)	Streptozotocin-induced diabetic Sprague-Dawley rats	Melatonin significantly enhanced retinal antioxidant capacity by increasing GSH levels and upregulating GCL expression, while simultaneously reducing oxidative stress through lowering ROS and lipid peroxidation. It also exerted strong anti-inflammatory effects, decreasing levels of TNF-α, IL-1β, and iNOS in diabetic retinal tissue. Functionally, melatonin preserved retinal integrity by improving ERG a- and b-wave amplitudes and reducing the extent of retinal degeneration, collectively demonstrating robust structural and functional protection of the retina	Melatonin acts through activation of the PI3K/Akt–Nrf2 pathway, promoting the nuclear localization of Nrf2 and increasing transcription of key antioxidant genes such as GCL. At the same time, it inhibits the NF-κB inflammatory pathway, reducing the transcription of proinflammatory cytokines. Overall, this combined enhancement of antioxidant defense and suppression of inflammatory signaling enables melatonin to protect retinal neurons and vascular tissue in diabetic retinopathy
Testes	González-Arto et al. (64)	Ram testis (and other reproductive tract tissues)	The key findings: RT-PCR detected mRNA for AANAT and ASMT in all studied organs; qPCR and Western blot showed significantly higher expression of these enzymes in testis (*P* < 0.05); immunohistochemistry localized AANAT and ASMT in Leydig cells, spermatocytes and spermatids in the testis; measurable melatonin levels found in testicular tissue and tail of epididymis; seminal plasma melatonin higher in ejaculate collected at 0600 than at 1000 or 1400. The authors conclude that the testes are likely a source of the high melatonin levels in ram seminal plasma, and that locally produced melatonin may act in an autocrine/paracrine manner in the testis	Expression of melatonin-synthesizing enzymes (AANAT, ASMT) in Leydig cells, spermatocytes and spermatids → testis as source of local melatonin; immunolocalization suggests paracrine/autocrine role
	Xu et al. (65)	Rooster (in vivo) and isolated Leydig cells (in vitro)	Prolonged light (16 h) increased testosterone and decreased testicular melatonin and receptor/enzyme expression. In cultured Leydig cells, melatonin (0.1–100 ng/mL) inhibited testosterone synthesis; silencing of MEL1A/B attenuated this inhibition. Melatonin reduced intracellular cAMP and p-CREB; stimulating the cAMP/PKA pathway reversed melatonin’s inhibition. → Concludes that photoperiod increases testosterone in roosters partly by suppressing the local melatonin system in the testis, and melatonin inhibits testosterone synthesis via the MEL1A/B-cAMP-PKA-CREB pathway	Longer photoperiod → ↓ testicular melatonin production (↓ AANAT, ↓ MEL1A / MEL1B receptors) → ↑ testosterone; melatonin treatment in vitro inhibited testosterone via MEL1A/B → ↓cAMP → ↓ PKA/CREB signaling in Leydig cells
	Rossi et al. (66)	Human infertile men (testicular biopsy samples) and in vitro cultured human and hamster macrophage/mast cell line	In human biopsies, lower melatonin levels are associated with higher macrophage infiltration and higher inflammatory cytokines; higher melatonin levels are associated with increased SOD1, peroxiredoxin-1, and catalase In vitro, melatonin suppressed macrophage proliferation and pro-inflammatory marker expression; in mast cells increased antioxidant enzyme expression and reduced ROS. → Suggests local anti-inflammatory + antioxidant role of melatonin in human testis	Melatonin inversely correlated with macrophage infiltration, TNF-α, IL-1β, COX-2; positively correlated with antioxidant enzymes (SOD, peroxiredoxin-1, and catalase). In vitro: melatonin inhibited proliferation and cytokine/COX2 expression in macrophages; increased antioxidant enzyme expression and decreased ROS in mast cells
Liver and metabolic tissues	Kato et al. (67)	Mouse 3T3-L1 preadipocyte cell line	Melatonin significantly increased PPARγ expression, promoted differentiation to adipocytes, induced smaller lipid droplets, upregulated lipolysis markers (ATGL, perilipin, CGI-58), promoted mitochondrial biogenesis (↑ PGC-1α, NRF-1, TFAM), increased adiponectin secretion and its receptors	Melatonin effect on adipogenesis, mitochondrial biogenesis, lipolysis, and adiponectin secretion
	Heo et al. (68)	Human HepG2 hepatocyte cell line + HFD mice	In palmitic acid-treated HepG2 cells, palmitate decreased p-AKT and increased *FETUA*; melatonin restored p-AKT and inhibited FETUA. In HFD mice, melatonin reduced body-weight gain, improved hepatic steatosis and insulin sensitivity, lowered FETUA and ER-stress markers	Hepatic insulin resistance/steatosis, via fetuin-A (AHSG) and ER stress, AKT phosphorylation
Bone	Zhang et al. (69)	In vitro: BMSCs (osteogenic differentiation assays); In vivo: OVX mice bone-loss model (female mice)	In vitro (BMSCs): melatonin increased osteogenic differentiation under osteoporotic conditions: ↑ osteoblast markers: Runx2, BMP2, ALP, OCN, OPN, BMP4, ↑ ALP activity and mineralization, ↓ ROS production and apoptosis, Activated HGF → ↓PTEN → ↑Wnt/β-catenin signaling. In vivo (OVX mice): Melatonin improved bone microstructure, ↑ BMD, ↑ BV/TV, Tb.N, Tb.Th, ↓ Tb.Sp, serum markers: ↑ BALP, ↓ TRAP5b. Mechanistic knockdown experiments confirmed HGF/PTEN/Wnt/β-catenin axis is necessary for melatonin’s osteogenic effect	Melatonin up-regulates HGF → down-regulates PTEN → activates Wnt/β-catenin → enhances osteogenic differentiation (↑Runx2, BMP2, OCN, OPN, BMP4) → improved bone microstructure and attenuated bone loss
	Sharan et al. (70)	Female mice: OVX plus WT and melatonin-receptor knockout (*MT1*^−/−^ and *MT2*^−/−^) models; in vivo bone structural and functional analysis	micro-CT bone microarchitecture (BV/TV, Tb.N, Tb.Sp, etc.), histomorphometry (osteoblast number, bone formation rate), serum markers (osteocalcin, CTX), gene expression assays in osteoblasts (proliferation/differentiation)	Exogenous melatonin acts via the MT2 receptor to enhance bone formation (osteoblast number/ activity) rather than altering bone resorption; thus, it counteracts estrogen deficiency-induced bone loss by increasing osteoblast number and bone formation rate
Cardiovascular/endothelial compartment	Zheng et al. (71)	HCAECs in vitro; THP-1/macrophages; CAWS-induced Kawasaki disease mouse model (in vivo)	Melatonin suppressed apoptosis in HCAECs via the MT/CREB → ATG3/autophagy axis and ameliorated vasculitis in CAWS mice; it also reduced macrophage cytokine production that would otherwise damage endothelial cells	Melatonin activates MT receptor → CREB → upregulates ATG3 and promotes autophagy, thereby reducing endothelial apoptosis (direct endothelial protection) and also reduces proinflammatory cytokines from macrophages (indirect paracrine benefit). This article explicitly connects melatonin receptor signaling to autophagy in vascular endothelial cells
Mitochondria	Suofu et al. (72)	Mouse/rat neuronal mitochondria + in vivo mouse ischemic brain injury	Demonstrated intracellular mitochondrial melatonin synthesis and a mitochondrial GPCR (MT1) signaling cascade that protects against ischemic injury	Mitochondria synthesize melatonin in the matrix → release and activate mitochondrial MT1 receptor → inhibit cytochrome c release and caspase activation (termed “automitocrine”)

AANAT, arylalkylamine *N-*acetyltransferase; Akt, protein kinase B; ALLN, *N*-acetyl-l-leucyl-l-leucinal; ALT, alkaline phosphatase; AMPK, adenosine monophosphate (AMP)-activated protein kinase; ASMT, acetylserotonin methyltransferase; AT, angiotensin; ATG2B, autophagy-related protein 2 homolog B; ATG3, autophagy gene; ATGL, adipose triglyceride lipase; BALP, bone alkaline phosphatase; BMD, bone mineral density; BM-DCs, bone marrow-derived dendritic cells; BMP, bone morphologic protein; BMPR1B, bone morphogenetic protein receptor type-1B; BMSCs, bone-marrow mesenchymal stem cells; BV/TV, trabecular bone volume fraction; cAMP, cyclin adenosine monophosphate; CAWS, Candida albicans Water-Soluble fraction; CGI-58, coactivator of ATGL; *CHOP*, CCAAT/enhancer-binding protein (EBP) homologous protein; Col-1, collagen 1; COX, cyclooxygenase; CT, computed tomography; D_4_ receptor; domapine type IV receptor; ER, endoplasmic reticulum; ERG, electroretinogram; ERK5, Extracellular signal-regulated kinase; FETUA, fetuin-A [alpha-2-HS-glycoprotein (AHSG)] gene; Fox1, forkhead box protein O1; GCL, glutamate-cysteine ligase; γ-GCS, γ-glutamylcysteine synthetase; GSH, glutathione; HCAECs, human coronary artery endothelial cells; HFD, high-fat diet; HIOMT, hydroxyindole-O-methyltransferase; HO-1, heme oxygenase-1; ICR, Institute for Cancer Research; IL, interleukin; iNOS, inducible nitric oxide synthase; IVF, in vitro fertilization; LC3, light-chain 3; LPS, lipopolysaccharide; MDA, malondialdehyde; MEL1A/B, Melatonin Receptor Type 1A (MTNR1A) and Type 1B (MTNR1B); MITF, microphthalmia-associated transcription factor; MT, melatonin receptor; mTOR, mechanistic target of rapamycin; NF-κB, nuclear factor kappa-light-chain-enhancer of activated B cells; NQ01, (NAD(P)H:quinone oxidoreductase 1; NRF2, nuclear factor erythroid 2-related factor 2; OCN, osteocalcin; 8-OHdG, 8-hydroxy-2′-deoxyguanosine; OHSS, ovarian hyperstimulation syndrome; OPN, osteopontin; OVX, ovariectomized; p-Akt, phosphorylated Akt; p-CREB, phosphorylated cyclic adenosine monophosphate response element-binding protein; PCOS, polycystic ovarian syndrome; PDTC, pyrrolidine dithiocarbamate; PGC-1α, peroxisome proliferator-activated receptor-gamma coactivator 1-alpha; PHA, phytohemagglutinin; PI3K, phosphatidylinositol 3-kinase; PKA, protein kinase A; PPARγ, peroxisome proliferator-activated receptor gamma; PTEN, phosphatase and tensin homolog; qPCR, quantitative polymerase chain reaction; ROS, reactive oxygen species; RT-PCR, reverse transcription polymerase chain reaction; SAP, severe acute pancreatitis; S6K1, p70 ribosomal S6 kinase 1; SESN2, sestrin 2; 4EBP1 (4E-binding protein 1); siRNA, small interfering ribonucleic acid; SIRT1, sirtuin 1; SIRT3, sirtuin 3; SOD, superoxide dismutase; Tb.N, trabecular number; Tb.Th, trabecular thickness; Tb.Sp, trabecular separation; TFAM, mitochondrial transcription factor A; THP, Tohoku Hospital Pediatrics; TNF-α, tumor necrosis factor-alpha; TPH, tryptophan hydroxylase; TRAP, tartrate-resistant acid phosphatase 5 b; UVB, ultraviolet-B radiation; WT, wild type.

#### Immune cells: autocrine melatonin as an immunoregulatory switch.

A prominent mechanistic work by Muxel et al. (40) showed that the TLR4/TRIF → IRF3 pathway causes AANAT expression in macrophages activated by LPS, which results in endogenous melatonin production that functions autocrinally to decrease M1 polarization and postpone apoptosis. The activation of NF-κB in RAW 264.7 macrophages transcriptionally upregulates AANAT, resulting in melatonin that enhances phagocytic function, which was reversed by blocking melatonin receptors.

Several integrative experiments have shown that extrapineal/autocrine melatonin converges on three recurrent routes across tissues: *1*) regulation of signaling pathways by local receptors, including PI3K/Akt, adenosine monophosphate (AMP)-activated protein kinase (AMPK), sirtuin 3 (SIRT3), nuclear factor erythroid 2-related factor 2 (Nrf2), and NF-κB; *2*) quality control and mitochondrial protection; and *3*) paracrine, vagal, and endocrine cross talk that links local systemic physiology to tissue states (73). Autocrine melatonin functions as a quick, tissue-specific cytoprotective and metabolic regulator, whereas pineal melatonin coordinates whole body circadian timing, according to recent reviews that highlight the idea of dual melatonin sources—pineal systemic chronobiotic output versus local stress-responsive production (29). These local roles are supported by experimental knockdown, knockout, and tissue-specific manipulation investigations in animal and cell models (74). A twin system for redox protection, metabolic tuning, and local-to-systemic communication is thus formed by extrapineal autocrine melatonin, a conserved, tissue-intrinsic protective mechanism that enhances pineal circadian signaling.

#### Ovarian autocrine/paracrine signaling.

Melatonin, which is produced locally in ovarian tissues, has been demonstrated in both in vitro and in vivo experiments to enhance *1*) antioxidant enzymes [e.g., superoxide dismutase (SOD), catalase], which lower reactive oxygen species (ROS) in follicular fluid; *2*) oocyte mitochondrial function and adenosine triphosphate (ATP) levels; and *2*) steroidogenic enzymes and progesterone secretion through receptor-dependent phosphoinositide-3 kinase (PI3K)/protein kinase B (Akt) and sirtuin (SIRT) signaling pathways (75). Melatonin protects against follicular cell damage by inhibiting serum starvation-induced autophagy in granulosa-like KGN cells through the activation of the PI3K/Akt/mechanistic target of rapamycin (mTOR) pathway (76). Furthermore, an in vitro maturation (IVM) study, which used immature human oocytes from older reproductive-aged women (≥35 yr) compared with younger women (<35 yr), supports the idea that melatonin reduces oxidative stress during maturation and preserves oocyte quality (77). When combined, these results suggest that melatonin has an autocrine role in ensuring follicular survival, promoting mitochondrial protection, preventing autophagy, protecting gametes, and promoting steroidogenesis through the PI3K/Akt/SIRT pathways.

#### Pineal gland.

Numerous studies show that local, intrapineal processes at the receptor, enzyme, and paracrine levels precisely control melatonin synthesis in the pineal gland. A unique receptor-receptor feedback modulation was revealed by González-Arto et al. (64), who demonstrated that D_4_ dopamine receptors form heteromers with adrenergic receptors (α_1_B-D_4_ and β_1_-D_4_) to suppress adrenergic stimulation of melatonin synthesis in a circadian-dependent way. Similarly, functional melatonin (MT_1_/MT_2_) receptors on pinealocytes that negatively control melatonin production were found by Lépinay et al. (13). Blocking these receptors increased cAMP and melatonin output, supporting an intrapineal autoinhibitory feedback loop.

The MT_1_/MT_2_ receptor signaling at the intracellular signaling level was examined, and a unique G-protein requirement for extracellular signal-regulated kinase (ERK) activation was observed, offering a molecular foundation for various local melatonin receptor signaling in pinealocytes (49). Nocturnal melatonin synthesis is further regulated by posttranslational control of the essential enzyme arylalkylamine *N*-acetyltransferase (AANAT) (51). Pineal indole production is also influenced by local paracrine regulation. The local renin-angiotensin system tonically regulates tryptophan hydroxylase activity and precursor availability, thereby modulating melatonin output, according to Baltatu et al. (52), who found that blocking AT1 receptors or lowering brain angiotensinogen decreased pineal serotonin and melatonin precursors.

Overall, these studies show that a network of intrapineal mechanisms, such as receptor heteromerization, autocrine feedback via MT receptors, enzyme stabilization via posttranslational modifications, and paracrine angiotensinergic signaling, regulates pineal melatonin synthesis in a way that is precisely circadian-dependent.

#### Cardiovascular/endothelial autocrine action.

Melatonin, which functions autocrinally to control vascular tone, lower oxidative stress, and maintain mitochondrial function, can be locally synthesized by endothelial cells and cardiomyocytes. According to experimental research, autocrine melatonin improves vasodilation by increasing nitric oxide (NO) bioavailability, shields mitochondria from oxidative damage, and stops endothelial dysfunction, which is a major contributing factor to the development of atherosclerosis and hypertension (78). Melatonin generated from endothelial cells has been demonstrated to enhance vascular relaxation in spontaneously hypertensive rats by lowering ROS and boosting NO availability. This lowers blood pressure and prevents early atherosclerotic alterations. According to these results, autocrine melatonin is a prospective therapeutic target for cardiovascular illnesses, such as hypertension and ischemic situations, and it plays a crucial role in preserving vascular homeostasis (78).

Long ago, the therapeutic potential of melatonin in cardiovascular functions was documented, and this was attributed to its antioxidant property (79). For instance, pinealectomy-induced elimination of circulating melatonin slowly, but persistently, increased rats’ arterial blood pressure (80, 81), and this effect was attenuated by melatonin (82). Similar findings were reported in aging humans, as the nighttime melatonin-producing capacity of the pineal gland declines with age (83, 84). It is noteworthy that there is normally a nocturnal drop in blood pressure in humans, which has been associated with the 24-h variation in melatonin levels (85). The antihypertensive effect of melatonin is mediated via MT1 and MT2, and the anterior hypothalamic area is thought to be the target site, considering its known involvement in blood pressure regulation (86, 87). Other known antihypertensive mechanisms of melatonin include the reduction of circulating catecholamine levels and the relaxation of the smooth muscle in the peripheral blood vessels (88).

#### Gut.

It is now known that enterochromaffin and other mucosal cells in the gut, which were earlier believed to produce huge quantity of melatonin and thus, not depend on pineal input, are not a major source of melatonin (89). According to experimental data, melatonin generated from the gut has both autocrine and paracrine effects on nearby tissues, strengthening tight junction integrity and antioxidant defenses to maintain barrier function (90). Melatonin directly regulates motility and secretion in animal models, including the stimulation of mucus and bicarbonate release, and pinealectomy studies confirm intestinal melatonin synthesis persists locally, highlighting a gut-intrinsic system (91). More recent research also shows a functional interaction with enteroendocrine signaling, where melatonin activates vagal afferent pathways and amplifies glucagon-like peptide 1 (GLP-1) release.

#### Skin and hair follicles.

Foundational works have shown that melatonin is produced from mammalian skin, including hamsters (7) and humans (6). To shield skin cells from oxidative damage, autocrine melatonin signaling through the cutaneous serotoninergic/melatoninergic system (5) is crucial. This system is part of the neuroimmunoendocrine organ, thereby positioning the skin as an organ that modulates homeostasis using mediators similar to the brain, immune, and endocrine systems (92). Nuclear factor erythroid 2-related factor 2 (NRF2) is triggered in human melanocytes by melatonin and its metabolites, such as *N*-acetyl-*N*-formyl-5-methoxykynuramine (AFMK), 6-hydroxymelatonin (6-OHM), *N*-acetylserotonin (NAS), and 5-methoxytryptamine (5-MT). Cellular protection against ultraviolet B (UVB)-induced oxidative damage is enhanced by this activation, which triggers phase 2 antioxidant enzymes, including glutathione peroxidase, catalase, and superoxide dismutase. It is noteworthy that these protective benefits happen without the need for traditional melatonin membrane receptors, indicating a direct intracellular mechanism (93).

Apart from providing antioxidant protection, melatonin and its byproducts lessen UV-induced DNA damage in skin cells. By phosphorylating p53 at Ser-15, a crucial stage in the DNA damage response, these substances promote DNA repair and reduce the production of cyclobutane pyrimidine dimers (CPDs), according to research on topical administration. Further shielding epidermal cells from genotoxic stress also improves nucleotide excision repair by encouraging interactions between damaged DNA and repair factors such as Xeroderma Pigmentosum Complementation Group C (XPC) and Xeroderma Pigmentosum Complementation Group A (XPA) (94).

Melatonin also controls the cycling of hair follicles and pigmentation. Through the Nrf2/PI3K/Akt pathway, it promotes the expression of microphthalmia-associated transcription factor (MITF) in melanocytes, which in turn modifies melanogenesis. In addition, melatonin stimulates the production of Wingless-type MMTV integration site family member 3A (Wnt3a) and Wnt5a in hair follicle stem cells, triggering the Wnt/β-catenin signaling pathway, which is essential for the growth and cycling of hair follicles (74). All of these investigations show that autocrine melatonin regulates local development and pigmentation programs and serves as the skin’s primary antioxidant and repair system.

#### Retina.

Numerous studies have shown that melatonin, which acts through both receptor-dependent and antioxidant/anti-inflammatory mechanisms, is essential for preserving retinal function and structural integrity. According to Baba et al. (61), wild-type mice showed greater scotopic and photopic electroretinogram (ERG) a- and b-wave amplitudes at night, whereas MT1 knockout (*MT1*^−/−^) mice lacked this diurnal difference, indicating that endogenous melatonin signaling via MT1 receptors is crucial for diurnal modulation of retinal function. Significant photoreceptor and ganglion cell loss was also observed in aging *MT1*^−^/^−^ animals, underscoring the significance of MT1-mediated melatonin signaling for retinal cell viability. In addition, melatonin inhibited NF-κB-mediated inflammation [including tumor necrosis factor-alpha (TNF-α), IL-1β, and inducible nitric oxide synthase (iNOS)] and triggered the PI3K/Akt-Nrf2 pathway, which encouraged Nrf2’s nuclear localization and the transcription of antioxidant genes, protecting retinal neurons and vascular tissue while maintaining ERG function (63). According to all of these studies, melatonin maintains retinal function and structural integrity through receptor-mediated diurnal signaling (MT1), as well as by controlling oxidative stress and inflammation, making it a potent neuroprotective agent in models of aging and diabetic retinopathy.

#### Testes.

The testes are both a location of melatonin synthesis and a target for melatonin’s autocrine and paracrine effects, according to a number of studies. According to González-Arto et al. (64), testicular tissue, the epididymal tail, and seminal plasma have detectable melatonin levels, and Leydig cells, spermatocytes, and spermatids of the ram testis express the essential melatonin-synthesizing enzymes AANAT and acetylserotonin methyltransferase (ASMT). Early in the morning, peak seminal plasma melatonin was found, indicating that locally generated melatonin may control testicular activity in an autocrine or paracrine way. Longer photoperiods boosted testosterone production in roosters while inhibiting testicular melatonin synthesis and receptor/enzyme expression, according to Xu et al. (65). By lowering intracellular cAMP and phosphorylated cyclic adenosine monophosphate response element-binding protein (CREB) levels via MEL1A/B receptors, melatonin suppressed testosterone synthesis in Leydig cells in vitro. This effect was reversible by activating the cAMP/PKA pathway. These results suggest that the MEL1A/B-cAMP-PKA-CREB signaling pathway is the mechanism by which local testicular melatonin inhibits steroidogenesis.

In human testicular biopsies, Rossi et al. (66) found that lower melatonin levels were linked to increased proinflammatory cytokines and macrophage infiltration, whereas greater melatonin was linked to enhanced production of antioxidant enzymes (SOD1, peroxiredoxin-1, and catalase). Melatonin may play a local protective role against inflammation and oxidative stress in the human testis, as evidenced by in vitro studies that reduced proinflammatory marker expression and macrophage proliferation while boosting antioxidant defenses and lowering reactive oxygen species in mast cells. All of these studies show that melatonin in the testis acts through autocrine, paracrine, and receptor-mediated mechanisms to regulate steroidogenesis locally and to protect against oxidative stress and inflammation.

#### Liver and metabolic tissues: hepatocyte/adipocyte autocrine roles.

Melatonin’s function in mitochondrial health and hepatocyte metabolism has been highlighted by recent research. In diabetic cardiomyopathy models, melatonin treatment enhanced Akt phosphorylation, reducing insulin resistance and indicating that the PI3K/Akt pathway is activated to enhance hepatocyte insulin sensitivity (95). In their study of melatonin’s potential as a treatment for liver illnesses, melatonin lowers inflammation and oxidative stress, shielding hepatocytes from lipotoxicity (96). The function of melatonin in mitochondria was further examined by a team that demonstrated that it can promote mitochondrial biogenesis and preserve the equilibrium of fusion and fission processes in metabolic tissues, such as the liver (67). Melatonin further improves glucose homeostasis by stimulating liver glycogen synthesis via the Protein Kinase C zeta (PKCζ)-Akt-GSK3β pathway (68, 97). Beyond glucose homeostasis, melatonin reduces hepatic steatosis and inflammation. For instance, it reduced hepatic lipid accumulation by alleviating lipopolysaccharide-induced Sterol Regulatory Element-Binding Protein (SREBP)-1c activation (98). Melatonin treatment reportedly improved liver enzymes and reduced gamma-glutamyl transferase activity, triglyceride levels, and concentrations of proinflammatory cytokines in patients with nonalcoholic steatohepatitis and nonalcoholic fatty liver disease (NAFLD) (68, 99, 100), possibly through attenuation of the alpha-2-HS-glycoprotein gene and its protein, fetuin-A (68). There is a possibility that melatonin will slow the progression of NAFLD to hepatic cancer, as recent studies have suggested nonalcoholic fatty pancreatic disease is a preneoplastic niche via metabolic and inflammatory pathways (101).

Melatonin also has important metabolic effects in adipose tissue. For instance, melatonin therapy improved insulin sensitivity and glucose absorption by increasing adiponectin secretion in adipocytes and GLUT4 translocation to the plasma membrane (102). The impact of melatonin on circadian rhythms, which can alter inflammatory pathways in adipose tissue and lessen inflammation in hypertrophic adipocytes, has been emphasized (103). All of these results point to the importance of autocrine melatonin signaling in liver and adipose tissues in preserving metabolic homeostasis through the promotion of insulin signaling, the inhibition of lipotoxicity, and the maintenance of mitochondrial health.

#### Bone.

Through a variety of processes, melatonin prevents bone loss and encourages osteogenic differentiation, by upregulating osteoblast markers [Runt-related transcription factor 2 (Runx2), Bone Morphogenetic Protein 2 (BMP2), alkaline phosphatase (ALP), osteocalcin (OCN), osteopontin (OPN), Bone Morphogenetic Protein 4 (BMP4)], increasing ALP activity and mineralization, decreasing ROS production and apoptosis, and activating the Hepatocyte Growth Factor (HGF) → Phosphatase and Tensin homolog (PTEN) → Wnt/β-catenin signaling pathway. Zhang et al. (69) showed in vitro that melatonin improved osteogenic differentiation of bone marrow mesenchymal stem cells (BMSCs) in osteoporotic conditions. Melatonin-treated ovariectomized (OVX) mice exhibited improved bone microstructure in vivo, including reduced trabecular separation (Tb.Sp), trabecular number (Tb.N), and trabecular thickness (Tb.Th); trabecular bone volume fraction (BV/TV); and enhanced bone mineral density (BMD). Serum indicators confirmed improved bone formation by showing lower TRAP5b and higher bone alkaline phosphatase (BALP). The crucial significance of the HGF/PTEN/Wnt/β-catenin axis in melatonin’s osteogenic impact was confirmed by mechanistic knockdown experiments.

In addition, Sharan et al. (70) demonstrated that MT2 receptors are the main mechanism by which exogenous melatonin promotes bone growth. Melatonin administration did not significantly impact bone resorption in OVX female mice, but it did enhance the number of osteoblasts and the rate of bone formation, as demonstrated by microcomputed tomography (mciro-CT) study of bone microarchitecture, histomorphometry, and serum markers [osteocalcin and C-terminal telopeptide of type I collagen (CTX)]. This suggests that melatonin primarily prevents bone loss caused by estrogen deprivation by promoting osteoblast activity and proliferation. All of these findings indicate that melatonin promotes osteoblast development and function through intracellular and receptor-mediated signaling pathways.

#### Mitochondria.

According to Suofu et al. (72), mitochondria can produce melatonin within the matrix and use it for autocrine-like signaling, or “automitocrine” signaling, to shield neurons from ischemia damage. Melatonin generated by the mitochondria stimulates mitochondrial MT1 receptors, which prevent apoptosis and lessen ischemic brain damage by inhibiting cytochrome c release and caspase activation. This work reveals a new intracellular function of melatonin in neuroprotection and mitochondrial function.

## DISCUSSION

According to a large and expanding body of research, melatonin is also locally synthesized in many peripheral tissues, including the gut, retina, skin, immune cells, ovary/testis, pancreatic islets, bone, and mitochondria-rich cells. Although melatonin is traditionally thought of as a pineal hormone that tells the body it is nighttime (4), melatonin frequently has autocrine or paracrine effects in various locations, meaning that the cell that produces it or neighboring cells react to it (104). In addition to using receptor-independent intracellular mechanisms (direct antioxidant/mitochondrial actions, modulation of sirtuins and redox sensors, and interactions with circadian transcriptional machinery), autocrine melatonin signaling also makes use of canonical G-protein-coupled receptors (MT_1_/MT_2_) and AhR expressed on the producing cells (4). These processes work together to enable tissue-specific tuning of cellular metabolism, redox balance, inflammatory tone, and local clocks by locally produced melatonin (104). The autocrine and paracrine actions mediated by these receptors, and also the receptor-independent actions, are not only elicited by melatonin but also by its metabolites. For instance, its metabolites like 6-hydroxymelatonin, 5-methoxytryptamine, 5-methoxytryptophol, 2-hydroxymelatonin, AFMK, and N^1^-acetyl-5-methoxykynuramine (AMK), have been implicated in photoreception, redox homeostasis, regulation of mitochondrial bioenergetics, antiapoptosis, and DNA repair, among others. Most of these functions are mediated by the metabolites via receptor-independent mechanisms (105, 106).

Although most body cells have their molecular clock machineries, the coherent timing of a whole body system is synchronized with the external light-dark cycle by the suprachiasmatic nucleus (SCN) of the hypothalamus, which receives light information via intrinsically photosensitive retinal ganglion cells and the retinohypothalamic tract (107, 108). Apart from melatonin, many hormones are known to show daily oscillation based on circadian rhythm, including sex steroids, glucocorticoids, thyroid-stimulating hormone, leptin, adiponectin, insulin, glucagon, ghrelin, growth hormone, and renin, among others (109). Although the pineal gland of cold-blooded vertebrates is photosensitive, this property is lost in higher vertebrates, who sense light with their inner retinal ganglion cells that then send neural signals to the brain’s visual areas and nonimage-forming areas like the pineal gland. Positive light signals elicit gamma-aminobutyric acid (GABA) secretion from the SCN, which inhibits the neurons that synapse with the hypothalamic paraventricular nucleus (PVN). This interrupts the signal to the pineal gland and consequently inhibits the synthesis of melatonin (109). Thus, interacting networks of clock genes in the SCN control the rhythm of melatonin production (110).

### Functional Consequences and Integrative Physiology

Available studies have demonstrated how melatonin integrates body functions. Pineal melatonin provides the body with night cues, whereas local melatonin in the gut supports barrier function and proper food processing following a meal (111). Autocrine melatonin enhances central neuroendocrine inputs in islets by regulating insulin release and β-cell resilience (104). Local melatonin quickly and locally reduces inflammation in immunological organs, avoiding systemic overactivation that could otherwise interfere with circadian metabolic routines. Thus, a hierarchical system is produced by this decentralized, tissue-autonomous melatonin signaling: extrapineal autocrine melatonin fine-tunes local physiological regulation to meet acute metabolic, oxidative, or immunological demands, whereas the pineal gland establishes a global temporal context (112). This architecture explains why melatonin’s therapeutic effects can occasionally be tissue-specific and why local melatonin synthesis (or its dysregulation) is linked to a variety of pathologies, from metabolic disease and inflammatory disorders to reproductive dysfunction and impaired wound healing.

### Gaps and Future Directions

Although various studies in recent times have enhanced our understanding of the physiological relevance of autocrine and paracrine melatonin signaling, some questions are yet to be adequately answered. Therefore, the following topics require more in-depth molecular and translational work: *1*) melatonin production and receptor expression quantitatively mapped across cell types and disease states, *2*) separating autocrine processes in vivo that are receptor-dependent from those that are receptor-independent, including dynamics of mitochondrial uptake, *3*) figuring out how nutrition, inflammation, microbiome, and tissue clocks affect local melatonin production, *4*) therapeutic targeting: is it possible and advantageous to increase or mimic local (autocrine) melatonin in tissue-specific disease?

### Comparison with Other Studies

This review highlights autocrine melatonin in contrast to broad overviews that treat melatonin primarily as a systemic, pineal-derived chronobiotic signal. It also demonstrates the unique, frequently noncircadian physiological roles of autocrine/paracrine production at extrapineal sites (gut, immune cells, reproductive organs, mitochondria, etc.). The majority of previous assessments either addressed melatonin’s antioxidant and sleep-regulating qualities generally or focused on pineal production and circadian signaling (nighttime endocrine melatonin). For instance, pineal-centric evaluations place a strong emphasis on circadian endocrine functions (such as sleep-wake regulation and the systemic night signal), but they do not methodically break down the intracellular signaling pathways or local autocrine/paracrine roles in peripheral tissues. Our investigation follows tissue-specific methods and intentionally distinguishes between extrapineal (autocrine/paracrine/mitochondrial) and pineal (endocrine) melatonin (113). Studies on mitochondrial melatonin (72) offer groundbreaking proof that melatonin can be produced by the mitochondria themselves, and that it can work locally to maintain mitochondrial function and inhibit apoptotic signals. A key tenet of this review is that it changed the paradigm from a strictly pineal endocrine model to one in which the majority of cells can produce local, noncircadian melatonin with immediate autocrine/mitochondrial activities.

Previous reviews that focus on the gut and immunoendocrine systems explained extrapineal melatonin production (such as immune cells and gut epithelium) and implied its roles in barrier function, redox regulation, and local immune surveillance. However, they frequently failed to integrate particular signaling cascades and receptor-dependent versus receptor-independent (antioxidant/mitochondrial) effects across tissues (91). Thus, experimental studies mapping autocrine melatonin onto mitochondrial activities, sirtuin/SOD/Nrf2 pathways, receptor signaling (MT1/MT2), and downstream functional consequences in each tissue are compiled in our review. To put it briefly, this review goes beyond previous studies that established the existence and general functions of extrapineal melatonin by *1*) categorizing evidence by tissues, *2*) separating autocrine/paracrine/mitochondrial production from endocrine pineal output, and *3*) connecting those loci to particular intracellular mechanisms (e.g., local melatonin regulating mitochondrial ROS, modulating SIRT3, engaging MT1/MT2-Gαi signaling, and changing local gene expression).

### Methodological Limitations and Heterogeneity

Although local melatonin production and activities are supported by the majority of experimental data, direct clinical generalizability is limited by a number of persistent methodological limitations. One of such is model heterogeneity, as the reviewed studies used primary cells, isolated cell lines, various animal models, and a small amount of human tissue. Second, cross-study comparisons are made more difficult by variations in species, cell type, and experimental settings (such as timing, substrate availability, and stressors). Third, the distinction between constitutive/local mitochondrial synthesis and traditional circadian pineal secretion is not always clear in research; experimental designs need to account for feeding/fasting conditions and time of day. Fourth, many studies did not distinguish receptor-dependent versus receptor-independent actions, as melatonin functions as an antioxidant/mitochondrial modulator directly and through MT1/MT2 G-protein coupled receptors (GPCRs), and not all research used mitochondrial-targeting tests, receptor antagonists, or knockdown/knockout models. Another limitation of this study is that quantification and localization of melatonin synthesis were heterogeneous in the reviewed studies, as many of them inferred local production from gene expression of biosynthetic enzymes rather than directly measuring melatonin in subcellular compartments. Finally, there were limited human translational data, and the majority of the evidence was preclinical, meaning that there are still few clinical studies and human tissue that directly evaluate autocrine melatonin processes.

### Implications for Clinical Practice and Translation

Treatments must take into account whether to target local autocrine/mitochondrial pools or systemic (pineal) melatonin rhythms. Local/mitochondrial melatonin enhances mitochondrial respiration, lowers ROS, and modifies sirtuin pathways (e.g., SIRT3). Autocrine melatonin produced by the gut or immune cells supports innate immune responses and barrier integrity (114). Local melatonin in reproductive tissues (placenta, testes, and ovary) maintains the quality of gametes and embryos and controls oxidative state (37). Though systemic melatonin supplementation at night has chronobiotic effects, to restore intracellular redox balance without changing the architecture of sleep, it may be necessary to increase local synthesis or administer melatonin that is targeted to the mitochondria (115). As multiple systems are influenced by melatonin, its systemic manipulation may have far-reaching consequences. Although safety is still unknown, methods that specifically increase local biosynthesis—such as gene therapy, small compounds that increase AANAT/ASMT expression, or formulations targeted at the mitochondria—may address metabolic problems and lessen off-target circadian effects in extrapineal sites.

### Conclusion

A layer of melatoninergic biology that is different from the traditional pineal endocrine signal and has biological and clinical significance is autocrine melatonin signaling in the pineal and numerous extrapineal locations. Preclinical research generally agrees on two complementary frameworks: *1*) receptor-mediated autocrine/paracrine signaling through MT1/MT2, which alters gene expression and local signaling pathways, and *2*) receptor-independent antioxidant/mitochondrial functions that maintain bioenergetic integrity. When combined, these processes enable melatonin to act as a homeostatic and local cytoprotective agent in a variety of tissues. There is translational potential, but before widespread clinical use, thorough mechanistic human research and better assays are required due to model heterogeneity and scarcity of human data. Future research will speed up safe, targeted translation by differentiating between endocrine and autocrine pools, measuring subcellular melatonin and using receptor/enzyme genetic models.

## SUPPLEMENTAL MATERIAL

Supplemental Files S1–S3: https://doi.org/10.6084/m9.figshare.30627593.

## References

[1] Reiter RJ, Rosales-Corral S, Tan DX, Jou MJ, Galano A, Xu B. Melatonin as a mitochondria-targeted antioxidant: one of evolution’s best ideas. Cell Mol Life Sci 74: 3863–3881, 2017. doi:10.1007/s00018-017-2609-7. 28864909 PMC11107735

[2] Back K. Melatonin metabolism, signaling and possible roles in plants. Plant J 105: 376–391, 2021. doi:10.1111/tpj.14915. 32645752

[3] Kim T-K, Slominski RM, Pyza E, Kleszczynski K, Tuckey RC, Reiter RJ, Holick MF, Slominski AT. Evolutionary formation of melatonin and vitamin D in early life forms: insects take centre stage. Biol Rev Camb Philos Soc 99: 1772–1790, 2024. doi:10.1111/brv.13091. 38686544 PMC11368659

[4] Reiter RJ, Tan DX, Fuentes-Broto L. Melatonin: a multitasking molecule. Prog Brain Res 181: 127–151, 2010. doi:10.1016/S0079-6123(08)81008-4. 20478436

[5] Slominski A, Wortsman J, Tobin DJ. The cutaneous serotoninergic/melatoninergic system: securing a place under the sun. FASEB J 19: 176–194, 2005. doi:10.1096/fj.04-2079rev. 15677341

[6] Slominski A, Pisarchik A, Semak I, Sweatman T, Wortsman J, Szczesniewski A, Slugocki G, McNulty J, Kauser S, Tobin DJ, Jing C, Johansson O. Serotoninergic and melatoninergic systems are fully expressed in human skin. FASEB J 16: 896–898, 2002. doi:10.1096/fj.01-0952fje. 12039872

[7] Slominski A, Baker J, Rosano TG, Guisti LW, Ermak G, Grande M, Gaudet SJ. Metabolism of serotonin to n-acetylserotonin, melatonin, and 5-methoxytryptamine in hamster skin culture. J Biol Chem 271: 12281–12286, 1996. doi:10.1074/jbc.271.21.12281. 8647827

[8] Edemann-Callesen H, Andersen HK, Ussing A, Virring A, Jennum P, Debes NM, Laursen T, Baandrup L, Gade C, Dettmann J, Holm J, Krogh C, Birkefoss K, Tarp S, Händel MN. Use of melatonin in children and adolescents with idiopathic chronic insomnia: a systematic review, meta-analysis, and clinical recommendation. EClinicalMedicine 61: 102048, 2023. doi:10.1016/j.eclinm.2023.102048. 37457117 PMC10339205

[9] Korkmaz A, Reiter RJ, Topal T, Manchester LC, Oter S, Tan DX. Melatonin: an established antioxidant worthy of use in clinical trials. Mol Med 15: 43–50, 2009. doi:10.2119/molmed.2008.00117. 19011689 PMC2582546

[10] Rurangwa J, Twambaze FM, Kagisha V, Alagbonsi AI. Rwandan coffee arabica has caffeine increasing with altitude, but undetectable phytomelatonin. Food Chem Adv 7: 100992, 2025. doi:10.1016/j.focha.2025.100992.

[11] Mayo JC, Sainz RM, González-Menéndez P, Hevia D, Cernuda-Cernuda R. Melatonin transport into mitochondria. Cell Mol Life Sci 74: 3927–3940, 2017. doi:10.1007/s00018-017-2616-8. 28828619 PMC11107582

[12] Jockers R, Delagrange P, Dubocovich ML, Markus RP, Renault N, Tosini G, Cecon E, Zlotos DP. Update on melatonin receptors: IUPHAR Review 20. Br J Pharmacol 173: 2702–2725, 2016. doi:10.1111/bph.13536. 27314810 PMC4995287

[13] Lépinay J, Taragnat C, Dubois J-P, Chesneau D, Jockers R, Delagrange P, Bozon V. Negative regulation of melatonin secretion by melatonin receptors in ovine pinealocytes. PLoS One 16: e0255249, 2021. doi:10.1371/journal.pone.0255249. 34324562 PMC8320996

[14] Macchi MM, Bruce JN. Human pineal physiology and functional significance of melatonin. Front Neuroendocrinol 25: 177–195, 2004. doi:10.1016/j.yfrne.2004.08.001. 15589268

[15] Slominski AT, Kim T-K, Slominski RM, Song Y, Qayyum S, Placha W, Janjetovic Z, Kleszczyński K, Atigadda V, Song Y, Raman C, Elferink CJ, Hobrath JV, Jetten AM, Reiter RJ. Melatonin and its metabolites can serve as agonists on the aryl hydrocarbon receptor and peroxisome proliferator-activated receptor Gamma. Int J Mol Sci 24: 15496, 2023. doi:10.3390/ijms242015496. 37895177 PMC10607054

[16] Chen C-Q, Fichna J, Bashashati M, Li Y-Y, Storr M. Distribution, function and physiological role of melatonin in the lower gut. World J Gastroenterol 17: 3888–3898, 2011. doi:10.3748/wjg.v17.i34.3888. 22025877 PMC3198018

[17] Tao J-L, Zhang X, Zhou J-Q, Li C-Y, Yang M-H, Liu Z-J, Zhang L-L, Deng S-L, Zhang L, Shen M, Liu G-S, Liu H-L. Melatonin alleviates hypoxia-induced apoptosis of granulosa cells by reducing ROS and activating MTNR1B–PKA–caspase8/9 pathway. Antioxidants (Basel) 10: 184, 2021. doi:10.3390/antiox10020184. 33525391 PMC7911142

[18] Namboodiri MAA, Valivullah HM, Moffett JR. Regulation of melatonin synthesis in the ovine pineal gland. Adv Exp Med Biol 294: 137–148, 1991. doi:10.1007/978-1-4684-5952-4_12. 1772063

[19] Olayaki L, Alagbonsi A, Adamson M, Ayodele O, Olawepo A. Melatonin administration to castrated rats reversed the castration-induced dyslipidemia while potentiating increased testosterone production from other nontesticular sources. Niger J Exp Clin Biosci 4: 6, 2016. doi:10.4103/njecp.njecp_1_16.

[20] Alagbonsi IA, Olayaki LA, Salman TM. Melatonin and vitamin C exacerbate Cannabis sativa -induced testicular damage when administered separately but ameliorate it when combined in rats. J Basic Clin Physiol Pharmacol 27: 277–287, 2016. doi:10.1515/jbcpp-2015-0061. 26479341

[21] Alagbonsi IA, Olayaki LA. Role of oxidative stress in Cannabis sativa -associated spermatotoxicity: evidence for ameliorative effect of combined but not separate melatonin and vitamin C. Middle East Fertil Soc J 22: 136–144, 2017. doi:10.1016/j.mefs.2016.12.004.

[22] Olayaki LA, Alagbonsi IA, Abdulkadir HO, Idowu FO. Low dose of melatonin ameliorates cryptorchidism-induced spermatotoxicity in rats. J Anat Soc India 66: 67–71, 2017. doi:10.1016/j.jasi.2017.05.010.

[23] Alagbonsi IA, Olayaki LA. Melatonin attenuates Δ9-tetrahydrocannabinol-induced reduction in rat sperm motility and kinematics in-vitro. Reprod Toxicol 77: 62–69, 2018. doi:10.1016/j.reprotox.2018.02.005. 29454037

[24] Olayaki LA, Alagbonsi IA, Abdulrahim AH, Adeyemi WJ, Bakare M, Omeiza N. Melatonin prevents and ameliorates lead-induced gonadotoxicity through antioxidative and hormonal mechanisms. Toxicol Ind Health 34: 596–608, 2018. doi:10.1177/0748233718773508. 29759042

[25] Alagbonsi AI, Olayaki LA, Abdulrahim HA, Suleiman MT, Bojuwade I, Omeiza NA, Sulaiman SO. Melatonin ameliorates ketoconazole-induced increase in thyroid function. RJMHS 3: 3–10, 2020. doi:10.4314/rjmhs.v3i1.2.

[26] Maliki OO, Alagbonsi AI, Ibitoye CM, Olayaki LA. Melatonin and Vitamin C modulate cassava diet-induced alteration in reproductive and thyroid functions. Niger J Exp Clin Biosci 9: 133–143, 2021. doi:10.4103/njecp.njecp_9_21.

[27] Omeiza NA, Abdulrahim HA, Alagbonsi AI, Ezurike PU, Soluoku TK, Isiabor H, Alli‐oluwafuyi AA. Melatonin salvages lead‐induced neuro‐cognitive shutdown, anxiety, and depressive‐like symptoms via oxido‐inflammatory and cholinergic mechanisms. Brain Behav 11: e2227, 2021. doi:10.1002/brb3.2227. 34087957 PMC8413791

[28] Abdulrahim HA, Alagbonsi IA, Amuda O, Omeiza NA, Feyitimi ARA, Olayaki LA. Cannabis sativa and/or melatonin do not alter brain lipid but alter oxidative mechanisms in female rats. J Cannabis Res. 3: 38, 2021. doi:10.1186/s42238-021-00095-9. 34412689 PMC8377844

[29] Zhang X, Yuan X-Q, Zhang X-M, Zhang XM. Melatonin reduces inflammation in intestinal cells, organoids and intestinal explants. Inflammopharmacology 29: 1555–1564, 2021 [Erratum in *Inflammopharmacology* 30: 685, 2022]. doi:10.1007/s10787-021-00869-w. 34431007

[30] Page MJ, McKenzie JE, Bossuyt PM, Boutron I, Hoffmann TC, Mulrow CD, Shamseer L, Tetzlaff JM, Akl EA, Brennan SE, Chou R, Glanville J, Grimshaw JM, Hróbjartsson A, Lalu MM, Li T, Loder EW, Mayo-Wilson E, McDonald S, McGuinness LA, Stewart LA, Thomas J, Tricco AC, Welch VA, Whiting P, Moher D. The PRISMA 2020 statement: an updated guideline for reporting systematic reviews. BMJ 372: n71, 2021. doi:10.1136/bmj.n71. 33782057 PMC8005924

[31] Hooijmans CR, Rovers MM, de Vries RB, Leenaars M, Ritskes-Hoitinga M, Langendam MW. SYRCLE’s risk of bias tool for animal studies. BMC Med Res Methodol 14: 43, 2014. doi:10.1186/1471-2288-14-43. 24667063 PMC4230647

[32] Nejadghaderi SA, Balibegloo M, Rezaei N. The Cochrane risk of bias assessment tool 2 (RoB 2) versus the original RoB: a perspective on the pros and cons. Health Sci Rep 7: e2165, 2024. doi:10.1002/hsr2.2165. 38835932 PMC11147813

[33] Zheng M, Liu M, Zhang C. Melatonin ameliorates ovarian hyperstimulation syndrome (OHSS) through SESN2 regulated antiapoptosis. Obstet Gynecol Int 2023: 1121227, 2023. doi:10.1155/2023/1121227. 37937274 PMC10626722

[34] Yu K, Wang R-X, Li M-H, Sun T-C, Zhou Y-W, Li Y-Y, Sun L-H, Zhang B-L, Lian Z-X, Xue S-G, Liu Y-X, Deng S-L. Melatonin reduces androgen production and upregulates heme oxygenase-1 expression in granulosa cells from PCOS patients with hypoestrogenia and hyperandrogenia. Oxid Med Cell Longev 2019: 8218650, 2019. doi:10.1155/2019/8218650. 31772710 PMC6854986

[35] Liu Y-F, Liu Z-Y, Li W-T, Wang P, Wang X-Y, Di R, He X-Y, Chu M-X. Effect of melatonin on ATG2B ‐mediated autophagy regulation in sheep granulosa cells with different Fec B genotypes. J Pineal Res 75: e12890, 2023. doi:10.1111/jpi.12890. 37226314

[36] Liu Y, Zhu X, Wu C, Lang Y, Zhao W, Li Y. Melatonin protects against ovarian damage by inhibiting autophagy in granulosa cells in rats. Clinics (Sao Paulo) 77: 100119, 2022. doi:10.1016/j.clinsp.2022.100119. 36194922 PMC9531038

[37] Tamura H, Kawamoto M, Sato S, Tamura I, Maekawa R, Taketani T, Aasada H, Takaki E, Nakai A, Reiter RJ, Sugino N. Long‐term melatonin treatment delays ovarian aging. J Pineal Res 62: 2017, e12381. doi:10.1111/jpi.12381. 27889913

[38] Zhang L, Zhang Z, Wang J, Lv D, Zhu T, Wang F, Tian X, Yao Y, Ji P, Liu G. Melatonin regulates the activities of ovary and delays the fertility decline in female animals via MT1/AMPK pathway. J Pineal Res 66: e12550, 2019. doi:10.1111/jpi.12550. 30597622

[39] Xu G, Dong Y, Wang Z, Ding H, Wang J, Zhao J, Liu H, Lv W. Melatonin attenuates oxidative stress-induced apoptosis of bovine ovarian granulosa cells by promoting mitophagy via SIRT1/FoxO1 signaling pathway. Int J Mol Sci 24: 12854, 2023. doi:10.3390/ijms241612854. 37629033 PMC10454225

[40] Muxel SM, Pires-Lapa MA, Monteiro AWA, Cecon E, Tamura EK, Floeter-Winter LM, Markus RP. NF-κB drives the synthesis of melatonin in RAW 264.7 macrophages by inducing the transcription of the arylalkylamine-n-acetyltransferase (AA-NAT) gene. PLoS One 7: e52010, 2012. doi:10.1371/journal.pone.0052010. 23284853 PMC3528721

[41] Xu M-M, Kang J-Y, Ji S, Wei Y-Y, Wei S-L, Ye J-J, Wang Y-G, Shen J-L, Wu H-M, Fei G-H. Melatonin suppresses macrophage M1 polarization and ROS‐mediated pyroptosis via activating ApoE/LDLR pathway in influenza A‐induced acute lung injury. Oxid Med Cell Longev 2022: 2520348, 2022. doi:10.1155/2022/2520348. 36425057 PMC9681554

[42] Sun S, Tang T, Wei M. Melatonin enhances the ability of M2 macrophages to secrete IL10 by inhibiting Erk5 signaling pathway. Mol Immunol. 162: 45–53, 2023. doi:10.1016/j.molimm.2023.08.009. 37647773

[43] Hu Y, Xiong Y, Zha K, Tao R, Chen L, Xue H, Yan C, Lin Z, Endo Y, Cao F, Zhou W, Liu G. Melatonin promotes BMSCs osteoblastic differentiation and relieves inflammation by suppressing the NF-κB pathways. Stem Cells Int 2023: 7638842, 2023. doi:10.1155/2023/7638842. 37274021 PMC10232925

[44] Carrillo‐Vico A, Calvo JR, Abreu P, Lardone PJ, García‐Mauriño S, Reiter RJ, Guerrero JM. Evidence of melatonin synthesis by human lymphocytes and its physiological significance: possible role as intracrine, autocrine, and/or paracrine substance. FASEB J 18: 537–539, 2004. doi:10.1096/fj.03-0694fje. 14715696

[45] Jiménez-Delgado A, Ortiz GG, Delgado-Lara DL, González-Usigli HA, González-Ortiz LJ, Cid-Hernández M, Cruz-Serrano JA, Pacheco-Moisés FP. Effect of melatonin administration on mitochondrial activity and oxidative stress markers in patients with Parkinson’s disease. Oxid Med Cell Longev 2021: 5577541, 2021. doi:10.1155/2021/5577541. 34707777 PMC8545577

[46] Lardone PJ, Carrillo-Vico A, Naranjo MC, De Felipe B, Vallejo A, Karasek M, Guerrero JM. Melatonin synthesized by Jurkat human leukemic T cell line is implicated in IL‐2 production. J Cell Physiol 206: 273–279, 2006. doi:10.1002/jcp.20461. 16021634

[47] Pires-Lapa MA, Carvalho-Sousa CE, Cecon E, Fernandes PA, Markus RP. β-Adrenoceptors trigger melatonin synthesis in phagocytes. Int J Mol Sci 19: 2182, 2018. doi:10.3390/ijms19082182. 30049944 PMC6121262

[48] González S, Moreno-Delgado D, Moreno E, Pérez-Capote K, Franco R, Mallol J, Cortés A, Casadó V, Lluís C, Ortiz J, Ferré S, Canela E, McCormick PJ. Circadian-related heteromerization of adrenergic and dopamine D_4_ receptors modulates melatonin synthesis and release in the pineal gland. PLoS Biol 10: e1001347, 2012. doi:10.1371/journal.pbio.1001347. 22723743 PMC3378626

[49] Chen M, Cecon E, Karamitri A, Gao W, Gerbier R, Ahmad R, Jockers R. Melatonin MT 1 and MT 2 receptor ERK signaling is differentially dependent on G i/o and G q/11 proteins. J Pineal Res 68: e12641, 2020. doi:10.1111/jpi.12641. 32080899

[50] Zatz M, Gastel JA, Heath JR, Klein DC. Chick pineal melatonin synthesis. J Neurochem 74: 2315–2321, 2000. doi:10.1046/j.1471-4159.2000.0742315.x. 10820191

[51] Obsil T, Ghirlando R, Klein DC, Ganguly S, Dyda F. Crystal structure of the 14-3-3ζ:serotonin *N-*acetyltransferase complex. Cell 105: 257–267, 2001. doi:10.1016/S0092-8674(01)00316-6. 11336675

[52] Baltatu O, Afeche SC, José dos Santos SH, Campos LA, Barbosa R, Michelini LC, Bader M, Cipolla-Neto J. Locally synthesized angiotensin modulates pineal melatonin generation. J Neurochem 80: 328–334, 2002. doi:10.1046/j.0022-3042.2001.00701.x. 11902123

[53] Sommansson A, Nylander O, Sjöblom M. Melatonin decreases duodenal epithelial paracellular permeability via a nicotinic receptor–dependent pathway in rats in vivo. J Pineal Res 54: 282–291, 2013. doi:10.1111/jpi.12013. 23009576

[54] Lin R, Wang Z, Cao J, Gao T, Dong Y, Chen Y. Role of melatonin in intestinal mucosal injury induced by restraint stress in mice. Pharm Biol 58: 342–351, 2020. doi:10.1080/13880209.2020.1750659. 32298156 PMC7178821

[55] Sun X, Shao Y, Jin Y, Huai J, Zhou Q, Huang Z, Wu J. Melatonin reduces bacterial translocation by preventing damage to the intestinal mucosa in an experimental severe acute pancreatitis rat model. Exp Ther Med 6: 1343–1349, 2013. doi:10.3892/etm.2013.1338. 24255660 PMC3829749

[56] Rungratanawanich W, LeFort KR, Cho Y, Li X, Song B. Melatonin prevents thioacetamide–induced gut leakiness and liver fibrosis through the gut–liver axis via modulating sirt1‐related deacetylation of gut junctional complex and hepatic proteins. J Pineal Res 76: e13007, 2024. doi:10.1111/jpi.13007. 39269018 PMC11480967

[57] Fernández-Gil B, Moneim AEA, Ortiz F, Shen Y-Q, Soto-Mercado V, Mendivil-Perez M, Guerra-Librero A, Acuña-Castroviejo D, Molina-Navarro MM, García-Verdugo JM, Sayed RKA, Florido J, Luna JD, López LC, Escames G. Melatonin protects rats from radiotherapy-induced small intestine toxicity. PLoS One 12: e0174474, 2017. doi:10.1371/journal.pone.0174474. 28403142 PMC5389624

[58] Shen Z, Miao K, Zhang X, Wu F, Zheng W, Cai L. Melatonin enhances the viability of random-pattern skin flaps by activating the NRF2 pathway. Arch Med Sci 21: 2144–2163, 2025. doi:10.5114/aoms/170172. 41403632 PMC12703607

[59] Sevilla A, Chéret J, Slominski RM, Slominski AT, Paus R. Revisiting the role of melatonin in human melanocyte physiology: a skin context perspective. J Pineal Res. 72: e12790, 2022. doi:10.1111/jpi.12790. 35133682 PMC8930624

[60] Bocheva G, Slominski RM, Janjetovic Z, Kim T-K, Böhm M, Steinbrink K, Reiter RJ, Kleszczyński K, Slominski AT. Protective role of melatonin and its metabolites in skin aging. Int J Mol Sci 23: 1238, 2022. doi:10.3390/ijms23031238. 35163162 PMC8835651

[61] Baba K, Pozdeyev N, Mazzoni F, Contreras-Alcantara S, Liu C, Kasamatsu M, Martinez-Merlos T, Strettoi E, Iuvone PM, Tosini G. Melatonin modulates visual function and cell viability in the mouse retina via the MT1 melatonin receptor. Proc Natl Acad Sci USA 106: 15043–15048, 2009. doi:10.1073/pnas.0904400106. 19706469 PMC2736407

[62] Atacak A, Baltaci SB, Akgun-Unal N, Mogulkoc R, Baltaci AK. Melatonin protects retinal tissue damage in streptozotocin-induced aged rats. Arch Gerontol Geriatr 112: 105035, 2023. doi:10.1016/j.archger.2023.105035. 37075585

[63] Jiang T, Chang Q, Cai J, Fan J, Zhang X, Xu G. Protective effects of melatonin on retinal inflammation and oxidative stress in experimental diabetic retinopathy. Oxid Med Cell Longev 2016: 3528274, 2016. doi:10.1155/2016/3528274. 27143993 PMC4837288

[64] González-Arto M, Aguilar D, Gaspar-Torrubia E, Gallego M, Carvajal-Serna M, Herrera-Marcos LV, Serrano-Blesa E, Hamilton TRDS, Pérez-Pé R, Muiño-Blanco T, Cebrián-Pérez JA, Casao A. Melatonin MT1 and MT2 receptors in the ram reproductive tract. Int J Mol Sci 18: 662, 2017. doi:10.3390/ijms18030662. 28335493 PMC5372674

[65] Xu G, Yuan Z, Hou J, Zhao J, Liu H, Lu W, Wang J. Prolonging photoperiod promotes testosterone synthesis of Leydig cells by directly targeting local melatonin system in rooster testes. Biol Reprod 105: 1317–1329, 2021. doi:10.1093/biolre/ioab155. 34401899

[66] Rossi SP, Windschuettl S, Matzkin ME, Terradas C, Ponzio R, Puigdomenech E, Levalle O, Calandra RS, Mayerhofer A, Frungieri MB. Melatonin in testes of infertile men: evidence for anti‐proliferative and anti‐oxidant effects on local macrophage and mast cell populations. Andrology 2: 436–449, 2014. doi:10.1111/j.2047-2927.2014.00207.x. 24659586

[67] Kato H, Tanaka G, Masuda S, Ogasawara J, Sakurai T, Kizaki T, Ohno H, Izawa T. Melatonin promotes adipogenesis and mitochondrial biogenesis in 3T3‐L1 preadipocytes. J Pineal Res 59: 267–275, 2015. doi:10.1111/jpi.12259. 26123001

[68] Heo J, Yoon DW, Yu JH, Kim NH, Yoo HJ, Seo JA, Kim SG, Choi KM, Baik SH, Choi DS, Kim NH. Melatonin improves insulin resistance and hepatic steatosis through attenuation of alpha-2-HS-glycoprotein. J Pineal Res 65: e12493, 2018. doi:10.1111/jpi.12493. 29607540

[69] Zhang J, Jia G, Xue P, Li Z. Melatonin restores osteoporosis-impaired osteogenic potential of bone marrow mesenchymal stem cells and alleviates bone loss through the HGF/PTEN/Wnt/β-catenin axis. Ther Adv Chronic Dis 12: 2040622321995685, 2021. doi:10.1177/2040622321995685. 34457228 PMC8392808

[70] Sharan K, Lewis K, Furukawa T, Yadav VK. Regulation of bone mass through pineal‐derived melatonin MT2 receptor pathway. J Pineal Res 63: e12423, 2017. doi:10.1111/jpi.12423. 28512916 PMC5575491

[71] Zheng Y, Huang S, Zhang J, Hou J, Wu F, Wang W, Han X, Gui Y. Melatonin alleviates vascular endothelial cell damage by regulating an autophagy‐apoptosis axis in Kawasaki disease. Cell Prolif 55: e13251, 2022. doi:10.1111/cpr.13251. 35582751 PMC9201377

[72] Suofu Y, Li W, Jean-Alphonse FG, Jia J, Khattar NK, Li J , et al Dual role of mitochondria in producing melatonin and driving GPCR signaling to block cytochrome c release. Proc Natl Acad Sci USA 114: E7997–E8006, 2017. doi:10.1073/pnas.1705768114. 28874589 PMC5617277

[73] Hardeland R. Redox biology of melatonin: discriminating between circadian and noncircadian functions. Antioxid Redox Signal 37: 704–725, 2022. doi:10.1089/ars.2021.0275. 35018802 PMC9587799

[74] Fischer TW, Slominski A, Tobin DJ, Paus R. Melatonin and the hair follicle. J Pineal Res 44: 1–15, 2008. doi:10.1111/j.1600-079X.2007.00512.x. 18078443

[75] Zheng B, Meng J, Zhu Y, Ding M, Zhang Y, Zhou J. Melatonin enhances SIRT1 to ameliorate mitochondrial membrane damage by activating PDK1/Akt in granulosa cells of PCOS. J Ovarian Res 14: 152, 2021. doi:10.1186/s13048-021-00912-y. 34758863 PMC8582167

[76] Wu D, Zhao W, Xu C, Zhou X, Leng X, Li Y. Melatonin suppresses serum starvation-induced autophagy of ovarian granulosa cells in premature ovarian insufficiency. BMC Womens Health 22: 474, 2022. doi:10.1186/s12905-022-02056-7. 36434569 PMC9700896

[77] Xi H, Huang L, Qiu L, Li S, Yan Y, Ding Y, Zhu Y, Wu F, Shi X, Zhao J, Chen R, Yao Q, Kou L. Enhancing oocyte in vitro maturation and quality by melatonin/bilirubin cationic nanoparticles: a promising strategy for assisted reproduction techniques. Int J Pharm X 8: 100268, 2024. doi:10.1016/j.ijpx.2024.100268. 39070171 PMC11278021

[78] Reiter RJ, Tan DX, Paredes SD, Fuentes-Broto L. Beneficial effects of melatonin in cardiovascular disease. Ann Med 42: 276–285, 2010. doi:10.3109/07853890903485748. 20455793

[79] Nava M, Quiroz Y, Vaziri N, Rodríguez-Iturbe B. Melatonin reduces renal interstitial inflammation and improves hypertension in spontaneously hypertensive rats. Am J Physiol Renal Physiol 284: F447–F454, 2003. doi:10.1152/ajprenal.00264.2002. 12441307

[80] Simko F, Paulis L. Melatonin as a potential antihypertensive treatment. J Pineal Res 42: 319–322, 2007. doi:10.1111/j.1600-079X.2007.00436.x. 17439547

[81] Zanoboni A, Zanoboni-Muciaccia W. Experimental hypertension in pinealectomized rats. Life Sci 6: 2327–2331, 1967. doi:10.1016/0024-3205(67)90043-4. 6060280

[82] Holmes SW, Sugden D. Proceedings: the effect of melatonin on pinealectomy-induced hypertension in the rat. Br J Pharmacol 56: 360P–361P, 1976. doi:10.1038/177791a0. 1260192 PMC1666943

[83] Sack RL, Lewy AJ, Erb DL, Vollmer WM, Singer CM. Human melatonin production decreases with age. J Pineal Res 3: 379–388, 1986. doi:10.1111/j.1600-079X.1986.tb00760.x. 3783419

[84] Reiter RJ. The pineal gland and melatonin in relation to aging: a summary of the theories and of the data. Exp Gerontol 30: 199–212, 1995. doi:10.1016/0531-5565(94)00045-5. 7556503

[85] Jonas M, Garfinkel D, Zisapel N, Laudon M, Grossman E. Impaired nocturnal melatonin secretion in non-dipper hypertensive patients. Blood Press 12: 19–24, 2003. doi:10.1097/00004872-200006001-00120. 12699131

[86] Xia C-M, Shao C-H, Xin L, Wang Y-R, Ding C-N, Wang J, Shen L-L, Li L, Cao Y-X, Zhu D-N. Melatonin on blood pressure in stress‐induced hypertension in rats. Clin Exp Pharmacol Physiol 35: 1258–1264, 2008. doi:10.1111/j.1440-1681.2008.05000.x. 18637016

[87] Wu Y-H, Zhou J-N, Balesar R, Unmehopa U, Bao A, Jockers R, Van Heerikhuize J, Swaab DF. Distribution of MT1 melatonin receptor immunoreactivity in the human hypothalamus and pituitary gland: colocalization of MT1 with vasopressin, oxytocin, and corticotropin‐releasing hormone. J Comp Neurol 499: 897–910, 2006. doi:10.1002/cne.21152. 17072839

[88] Ekmekcioglu C, Haslmayer P, Philipp C, Mehrabi MR, Glogar HD, Grimm M, Leibetseder VJ, Thalhammer T, Marktl W. Expression of the mt 1 melatonin receptor subtype in human coronary arteries. J Recept Signal Transduct Res 21: 85–91, 2001. doi:10.1081/RRS-100107144. 11693175

[89] Kennaway DJ. The mammalian gastro‐intestinal tract is a NOT a major extra‐pineal source of melatonin. J Pineal Res 75: e12906, 2023. doi:10.1111/jpi.12906. 37649458

[90] Ahmed R, Mahavadi S, Al-Shboul O, Bhattacharya S, Grider JR, Murthy KS. Characterization of signaling pathways coupled to melatonin receptors in gastrointestinal smooth muscle. Regul Pept 184: 96–103, 2013. doi:10.1016/j.regpep.2013.03.028. 23541890 PMC3748964

[91] Bubenik GA. Localization, physiological significance and possible clinical implication of gastrointestinal melatonin. Biol Signals Recept 10: 350–366, 2001. doi:10.1159/000046903. 11721091

[92] Slominski RM, Raman C, Jetten AM, Slominski AT. Neuro–immuno–endocrinology of the skin: how environment regulates body homeostasis. Nat Rev Endocrinol 21: 495–509, 2025 [Erratum in *Nat Rev Endocrinol* 21: 513, 2025]. doi:10.1038/s41574-025-01107-x. 40263492 PMC12239263

[93] Slominski AT, Hardeland R, Zmijewski MA, Slominski RM, Reiter RJ, Paus R. Melatonin: a cutaneous perspective on its production, metabolism, and functions. J Invest Dermatol 138: 490–499, 2018. doi:10.1016/j.jid.2017.10.025. 29428440 PMC5828910

[94] Janjetovic Z, Jarrett SG, Lee EF, Duprey C, Reiter RJ, Slominski AT. Melatonin and its metabolites protect human melanocytes against UVB-induced damage: involvement of NRF2-mediated pathways. Sci Rep 7: 1274, 2017. doi:10.1038/s41598-017-01305-2. 28455491 PMC5430855

[95] Watanabe K, Nakano M, Maruyama Y, Hirayama J, Suzuki N, Hattori A. Nocturnal melatonin increases glucose uptake via insulin-independent action in the goldfish brain. Front Endocrinol (Lausanne) 14: 1173113, 2023. doi:10.3389/fendo.2023.1173113. 37288290 PMC10242130

[96] Deng SL, Zhang BL, Reiter RJ, Liu YX. Melatonin ameliorates inflammation and oxidative stress by suppressing the p38MAPK signaling pathway in LPS-induced sheep orchitis. Antioxidants (Basel) 9: 1277, 2020. doi:10.3390/antiox9121277. 33327643 PMC7765110

[97] Shieh J, Wu H, Cheng K, Cheng J. Melatonin ameliorates high fat diet‐induced diabetes and stimulates glycogen synthesis via a PKCζ‐Akt‐GSK3β pathway in hepatic cells. J Pineal Res 47: 339–344, 2009. doi:10.1111/j.1600-079X.2009.00720.x. 19817973

[98] Chen X, Zhang C, Zhao M, Shi C-E, Zhu R-M, Wang H, Zhao H, Wei W, Li J-B, Xu D-X. Melatonin alleviates lipopolysaccharide-induced hepatic SREBP-1c activation and lipid accumulation in mice. J Pineal Res 51: 416–425, 2011. doi:10.1111/j.1600-079X.2011.00905.x. 21689150

[99] Gonciarz M, Gonciarz Z, Bielanski W, Mularczyk A, Konturek PC, Brzozowski T, Konturek SJ. The pilot study of 3-month course of melatonin treatment of patients with nonalcoholic steatohepatitis: effect on plasma levels of liver enzymes, lipids and melatonin. J Physiol Pharmacol 61: 705–710, 2010. doi:10.1016/s0016-5085(11)60476-9. 21224501

[100] Celinski K, Konturek PC, Slomka M, Cichoz-Lach H, Brzozowski T, Konturek SJ, Korolczuk A. Effects of treatment with melatonin and tryptophan on liver enzymes, parameters of fat metabolism and plasma levels of cytokines in patients with non-alcoholic fatty liver disease–14 months follow up. J Physiol Pharmacol 65: 75–82, 2014. doi:10.1111/j.1600-079x.2009.00677.x. 24622832

[101] Onohuean H, Nnolum-Orji NF, Naik Bukke SP, Abass KS, Alagbonsi AI, Choonara YE. Non-alcoholic fatty pancreas disease (NAFPD) as a pre-neoplastic niche: metabolic and inflammatory Gateways to pancreatic ductal adenocarcinoma. J Clin Transl Endocrinol 42: 100424, 2025. doi:10.1016/j.jcte.2025.100424. 41312164 PMC12648718

[102] Navarro-Alarcón M, Gil-Hernández F, Sánchez-González C, Llopis J, Villalón-Mir M, Olmedo P, Alarcón-Guijo P, Salagre D, Gaona L, Paredes M, Agil A. Melatonin improves levels of Zn and Cu in the muscle of diabetic obese rats. Pharmaceutics 13: 1535, 2021. doi:10.3390/pharmaceutics13101535. 34683825 PMC8539996

[103] de Farias TDSM, Cruz MM, de Sa RCDC, Severi I, Perugini J, Senzacqua M, Cerutti SM, Giordano A, Cinti S, Alonso-Vale MIC. Melatonin supplementation decreases hypertrophic obesity and inflammation induced by high-fat diet in mice. Front Endocrinol 10: 750, 2019. doi:10.3389/fendo.2019.00750. 31749764 PMC6848267

[104] Luchetti F, Canonico B, Betti M, Arcangeletti M, Pilolli F, Piroddi M, Canesi L, Papa S, Galli F. Melatonin signaling and cell protection function. FASEB J 24: 3603–3624, 2010. doi:10.1096/fj.10-154450. 20534884

[105] Slominski AT, Zmijewski MA, Semak I, Kim T-K, Janjetovic Z, Slominski RM, Zmijewski JW. Melatonin, mitochondria, and the skin. Cell Mol Life Sci 74: 3913–3925, 2017. doi:10.1007/s00018-017-2617-7. 28803347 PMC5693733

[106] Kim T-K, Kleszczynski K, Janjetovic Z, Sweatman T, Lin Z, Li W, Reiter RJ, Fischer TW, Slominski AT. Metabolism of melatonin and biological activity of intermediates of melatoninergic pathway in human skin cells. FASEB J 27: 2742–2755, 2013. doi:10.1096/fj.12-224691. 23620527 PMC3688757

[107] Partch CL, Green CB, Takahashi JS. Molecular architecture of the mammalian circadian clock. Trends Cell Biol 24: 90–99, 2014. doi:10.1016/j.tcb.2013.07.002. 23916625 PMC3946763

[108] Reppert SM, Weaver DR. Coordination of circadian timing in mammals. Nature 418: 935–941, 2002. doi:10.1038/nature00965. 12198538

[109] Begemann K, Rawashdeh O, Olejniczak I, Pilorz V, de Assis LVM, Osorio-Mendoza J, Oster H. Endocrine regulation of circadian rhythms. npj Biol Timing Sleep 2: 10, 2025. doi:10.1038/s44323-025-00024-6. 41775850 PMC12912344

[110] Takahashi JS, Hong HK, Ko CH, McDearmon EL. The genetics of mammalian circadian order and disorder: implications for physiology and disease. Nat Rev Genet 9: 764–775, 2008. doi:10.1038/nrg2430. 18802415 PMC3758473

[111] Acuña-Castroviejo D, Escames G, Venegas C, Díaz-Casado ME, Lima-Cabello E, López LC, Rosales-Corral S, Tan D-X, Reiter RJ. Extrapineal melatonin: sources, regulation, and potential functions. Cell Mol Life Sci 71: 2997–3025, 2014. doi:10.1007/s00018-014-1579-2. 24554058 PMC11113552

[112] Reiter RJ, Tan DX, Galano A. Melatonin: exceeding expectations. Physiology (Bethesda) 29: 325–333, 2014. doi:10.1152/physiol.00011.2014. 25180262

[113] Tan DX, Manchester L, Esteban-Zubero E, Zhou Z, Reiter R. Melatonin as a potent and inducible endogenous antioxidant: synthesis and metabolism. Molecules 20: 18886–18906, 2015. doi:10.3390/molecules201018886. 26501252 PMC6332205

[114] Paulose JK, Cassone VM. The melatonin-sensitive circadian clock of the enteric bacterium Enterobacter aerogenes. Gut Microbes 7: 424–427, 2016. doi:10.1080/19490976.2016.1208892. 27387841 PMC5154366

[115] Tan DX, Manchester L, Qin L, Reiter R. Melatonin: a mitochondrial targeting molecule involving mitochondrial protection and dynamics. Int J Mol Sci 17: 2124, 2016. doi:10.3390/ijms17122124. 27999288 PMC5187924

